# Cuproptosis in cancer: biological implications and therapeutic opportunities

**DOI:** 10.1186/s11658-024-00608-3

**Published:** 2024-06-25

**Authors:** Liping Li, Houfeng Zhou, Chenliang Zhang

**Affiliations:** 1 Department of Pharmacy, Chengdu Fifth People’s Hospital, Chengdu University of Traditional Chinese Medicine, Chengdu, Sichuan People’s Republic of China; 2 Division of Abdominal Cancer, Department of Medical Oncology, Cancer Center and Laboratory of Molecular Targeted Therapy in Oncology, West China Hospital, Sichuan University, Chengdu, 610041 Sichuan People’s Republic of China

**Keywords:** Copper, Cuproptosis, Tumorigenesis, Tumor therapy, Metabolism, Drug resistance

## Abstract

Cuproptosis, a newly identified copper (Cu)-dependent form of cell death, stands out due to its distinct mechanism that sets it apart from other known cell death pathways. The molecular underpinnings of cuproptosis involve the binding of Cu to lipoylated enzymes in the tricarboxylic acid cycle. This interaction triggers enzyme aggregation and proteotoxic stress, culminating in cell death. The specific mechanism of cuproptosis has yet to be fully elucidated. This newly recognized form of cell death has sparked numerous investigations into its role in tumorigenesis and cancer therapy. In this review, we summarized the current knowledge on Cu metabolism and its link to cancer. Furthermore, we delineated the molecular mechanisms of cuproptosis and summarized the roles of cuproptosis-related genes in cancer. Finally, we offered a comprehensive discussion of the most recent advancements in Cu ionophores and nanoparticle delivery systems that utilize cuproptosis as a cutting-edge strategy for cancer treatment.

## Introduction

Copper (Cu) is an important micronutrient in the human body that is vital in regulating various signaling pathways and associated biological processes, including mitochondrial respiration, detoxification of free radicals, and angiogenesis [1, 2]. In biological systems, copper exists in two oxidation states, i.e., divalent copper ion (Cu^2+^) and monovalent copper (Cu^+^). Imbalances in Cu homeostasis can contribute to the development of certain diseases, like Wilson’s disease caused by Cu overload and Menkes disease caused by Cu deficiency [2].

In the 1980s, it has been found that excessive Cu accumulation resulted in cell death [3]. Furthermore, Cu ionophores, which are lipid-soluble molecules that bind to Cu ions and transport them into cells, were discovered to induce cell death in tumor cells and have been used in clinical trials [4–6]. However, the molecular mechanism and specific form of cell death induced by Cu and Cu ionophores remained unclear for a long time. It was not until 2022 that Tsvetkov et al. unveiled a new form of cell death triggered by Cu called cuproptosis [7], which is independent of known forms of cell death like apoptosis, ferroptosis, autophagy, and necrosis. Regarding the molecular mechanism, Tsvetkov et al. found a strong association between cuproptosis and mitochondrial respiration and the lipoic acid (LA) pathway. The binding of Cu to components involved in lipoacylation in the tricarboxylic acid (TCA) cycle leads to their aggregation and downregulation of Fe–S cluster proteins, ultimately inducing proteotoxic stress and cell death [7]. Through genome-wide knockout screens and individual gene knockout studies, Tsvetkov et al. identified several key regulatory genes involved in cuproptosis [7].

Research has revealed the dichotomous role of Cu in tumorigenesis, progression, and therapeutic interventions. On one hand, elevated levels of Cu ions have been found to promote tumor growth, metastasis, and angiogenesis in various malignant tumors [8, 9], while on the other hand, excessive Cu ions can also induce tumor cell death [10, 11]. The discovery of cuproptosis has sparked interest among researchers exploring the relationship between cuproptosis and tumors. Since recent developments in this field of study are primarily focused on gene expression, one area of research involves investigating the expression levels of cuproptosis-related genes and their role in tumorigenesis, tumor treatment, and drug resistance to clarify the role of cuproptosis in these processes [12, 13]. Due to the unmet clinical need to treat cancer, new approaches are required. Another focus of the study is developing strategies for effective cancer treatment based on cuproptosis. Researchers have found that high levels of aerobic respiration and mitochondrial metabolism can sensitize tumor cells to cuproptosis, thereby enhancing their therapeutic effect [7]. The targeted delivery of Cu or Cu ionophores can be used to specifically kill cancer cells. In-depth research on cuproptosis in cancer will provide a scientific basis for developing clinical strategies aimed at targeting cuproptosis to improve tumor therapy.

In this review, we synthesized current understanding on copper metabolism and the molecular mechanisms underlying cuproptosis. We also explored the potential correlations between the expression of cuproptosis-associated genes across different tumor types and patient prognosis, with the goal of offering innovative insights into cuproptosis-based tumor therapy. Additionally, we outlined contemporary strategies for targeting cuproptosis in cancer treatment, including the use of Cu ionophores and nanoparticle-based precision delivery systems. Furthermore, we discussed the potential application of targeting cuproptosis to overcome resistance to chemotherapy in tumors. Our aim is to provide a new perspective for targeting cuproptosis-related tumor therapy.

## Cu metabolism

**Fig. 1:**
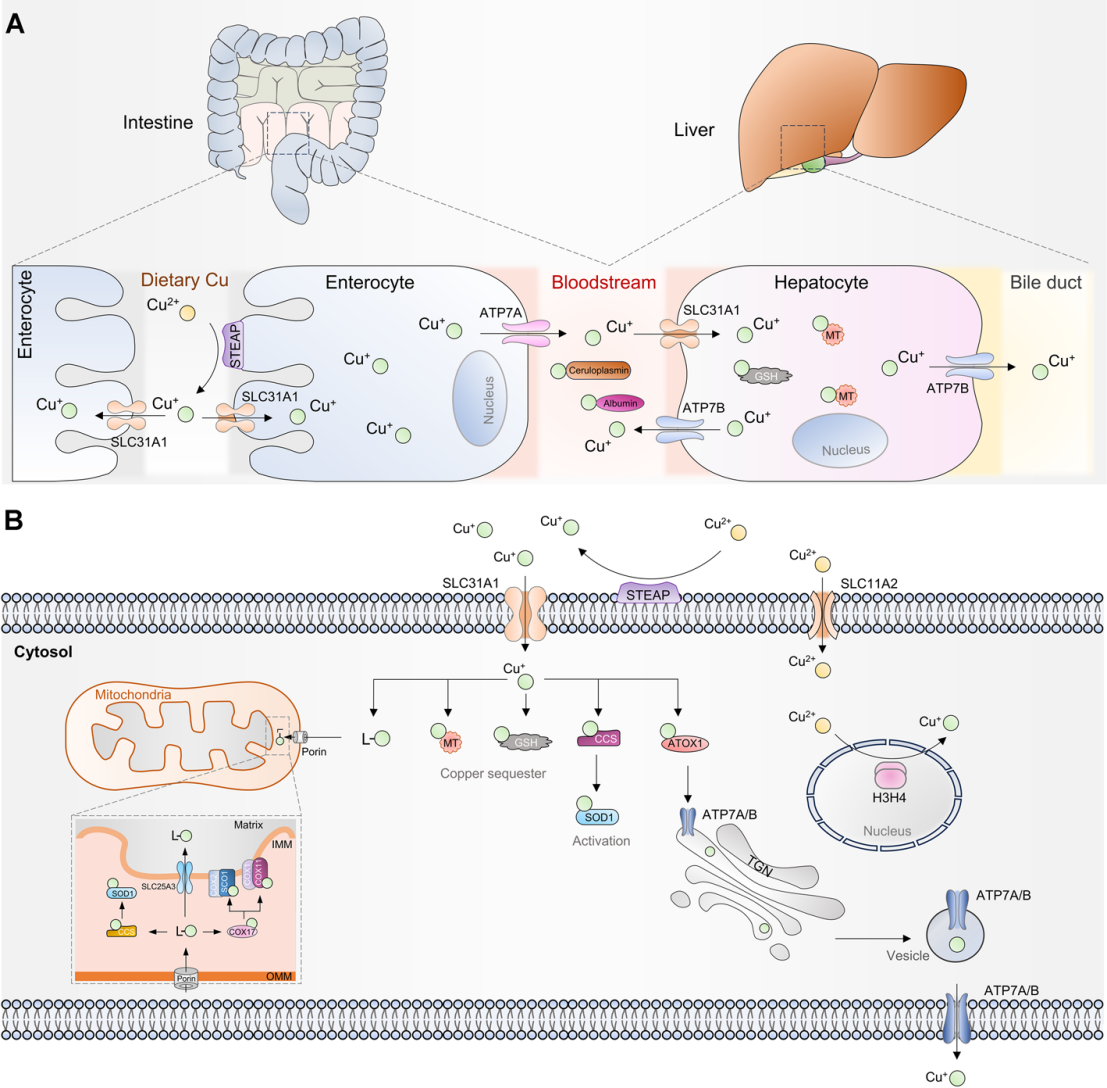
Schematic diagram of Cu metabolism. **A** Cu absorption and transport in the body. Cu is absorbed by the body through the small intestine and subsequently released into the bloodstream. Hepatocytes in the liver then absorb and store the Cu from the blood. These liver cells can secrete Cu into the blood or bile. **B** Cu trafficking in mammalian cells. Outside mammalian cells, Cu ions are transported into the cell by the Cu ion transporter SLC31A1. Once inside the cell, these ions can bind to Cu chaperones in the cytoplasm, such as GSH, MT, ATOX1, and CCS1. ATOX1 is responsible for delivering Cu ions to ATP7A/B and trafficking them outside the cell via the TGN-associated endo/exocytosis. CCS1 delivers Cu ions to SOD1. Ligand-bound Cu ions (L-Cu) in the cytoplasm are transported to the mitochondrial intermembrane space, where they can be delivered to SOD1 via CCS1 or to SCO1 and COX11 via COX17. Alternatively, they can be transported into the mitochondrial matrix via SLC25A3. H3/H4 in the nucleus can reduce Cu^2+^ to Cu^+^

Regulation of Cu levels in the body is crucial for maintaining normal cellular processes. Cu serves as a cofactor for numerous enzymes and is vital in various biochemical reactions. Cu is mainly obtained from diet, with visceral meat and shellfish being the richest sources. Once ingested, Cu ions are absorbed primarily in the duodenum and small intestine (Fig. 1A) [14]. Cu ions are taken up by the intestinal epithelium through a protein called Cu transporter 1 (CTR1), also known as solute carrier family 31 member 1 (SLC31A1) [15]. This protein is located on the apical side of enterocytes. The Cu absorption is facilitated by the activity of metalloreductases, such as six-transmembrane epithelial antigen of the prostate (STEAP) and duodenal cytochrome b [16, 17]. These enzymes reduce divalent Cu^2+^ to monovalent Cu^+^, facilitating absorption. After absorption, Cu is transported across the intestinal epithelium into the bloodstream. The ATPase Cu transporter α (ATP7A) protein, along with ATPase Cu transporter β (ATP7B), forms a Cu-transporting ATPase complex that plays a crucial role in Cu transport throughout the body. ATP7A is expressed in most tissues, while ATP7B is primarily expressed in the liver [18, 19]. These transporters are located in the trans-Golgi network (TGN) under normal physiological Cu levels. They pump Cu from the cytosol into the lumen of the TGN. However, when intracellular Cu levels increase, ATP7A and ATP7B translocate from the TGN to vesicular compartments within the cell. These vesicles eventually fuse with the plasma membrane, allowing Cu to be exported from the cell [20, 21]. This process ensures that excess Cu is removed from the cytosol and prevents Cu toxicity. ATP7B is vital for the export of Cu into the blood of hepatocytes. Cu is secreted back into the bloodstream by ATP7B and transported to various tissues and organs (Fig. 1A). Excess Cu is eliminated from the body through its export into bile, mediated by ATP7B [19, 20].

Cu ions in the bloodstream are typically bound to soluble chaperones such as albumin, macroglobulins, histidines, and transcopperin [22, 23]. In the cytoplasm, Cu can be stored by binding Cu chaperones such as metallothionein 1/2 (MT1/2) and glutathione (GSH) [20, 21, 24]. Moreover, cytosolic Cu can be bound by antioxidant 1 (ATOX1)—a critical Cu chaperone—and delivered to ATP7A or ATP7B located on the Golgi membrane in the TGN, ultimately secreted to the outside of the cell [25]. Furthermore, Cu can be stored in the mitochondria (Fig. 1B). Cu can enter the mitochondrial intermembrane space (IMS) through a nonproteinaceous anionic ligand (L-Cu), where it binds to cytochrome oxidase (COX) Cu chaperones and is delivered to other COXs or cuproenzymes [26]. Moreover, Cu^+^ can be transported into the mitochondrial matrix by the transmembrane transport protein, solute carrier family 25 member 3 (SLC25A3), located at the inner mitochondrial membrane (IMM) [27]. The Cu matrix can also be exported, but the transporter responsible for this has yet to be identified.

## Cuproptosis

Cu ionophores, such as elesclomol (ES) and disulfiram (DSF), are lipid-soluble compounds capable of reversibly binding to Cu ions and transporting them across the plasma or mitochondrial membrane, resulting in cell death [28, 29]. The precise mechanism remains unclear for a long time, but it is believed that Cu, rather than Cu ionophores, induces this cellular fatality [30, 31]. Initially, it was presumed that intracellular redox reactions and the resultant reactive oxygen species (ROS) were the mediators of cell death. ES amplifies oxidative stress and stimulates ROS production in tumor cells like melanoma [31], gynecological tumors [32], and lung cancer cells [33]. Similarly, DSF increased ROS levels in various tumor cells, including breast cancer [30, 34, 35], melanoma [36], gastric cancer [37], oral cavity squamous cell carcinoma [38], and hepatocellular carcinoma cells (HCC) [39]. Numerous research has reported that the ROS induced by these Cu ionophores combined with Cu mainly originate from mitochondria [6, 31, 32, 40, 41]. However, the role of ROS in Cu-induced cell death remains unclear. ROS scavengers, such as *N*-acetylcysteine (NAC), can mitigate the cell damage induced by Cu ionophores/Cu in certain cancer cells, including lung cancer cells [33, 42], gastric cancer [37], osteosarcoma [43], and melanoma [36]. Conversely, it has been reported that ROS scavengers do not exhibit the same protective effects in other cells, including breast cancer and glioblastoma cells [7, 32]. Therefore, there is still insufficient evidence to indicate that Cu-induced cell death is due to increased cellular ROS levels. Furthermore, the identification of Cu-mediated cell death has been controversial for a long time. Many studies reported it as different forms of cell death, including apoptosis [28, 36, 44–46], ferroptosis [47–51], autophagy [52, 53], and necrosis [49, 54].

Recently, a unique form of regulated cell death induced by intracellular Cu was identified by Tsvetkov et al. [7]. This process involves the aggregation of lipoylated mitochondrial enzymes and a reduction of Fe–S proteins, earning it the name “cuproptosis.” Notably, cell death does not occur when the Cu ionophore is devoid of Cu ions. This cell death can be counteracted by a Cu chelator but not by inhibitors of other known forms of cell death, including apoptosis, necroptosis, or ferroptosis [7]. Although the intracellular antioxidant and natural Cu chelator, GSH, could mitigate the toxicity of ES-Cu, other antioxidants such as NAC, JP4-039, ebselene, and α-tocopherol were ineffective in rescuing cells from the damage caused by ES-Cu [7], indicative of ROS-independent Cu-ionophore-mediated cuproptosis cell death. These findings suggest that cell death induced by Cu ionophores is a distinct, regulated form that is triggered by Cu, independent of known modes.

**Fig. 2:**
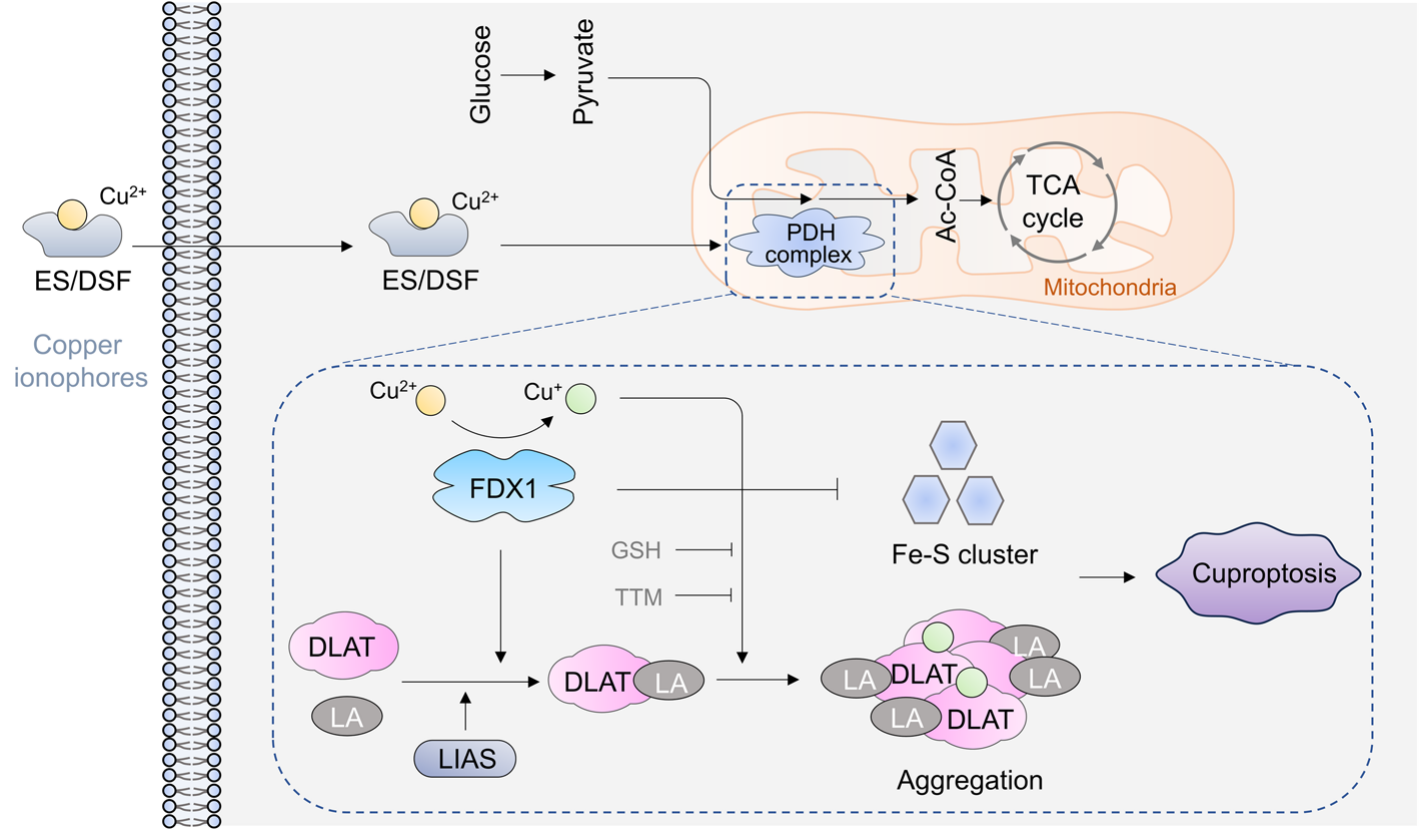
Schematic of the cuproptosis mechanism. Cu^+^ is transported into the cells by SLC31A1. Cu^2+^ is transported into cells by Cu ionophores, including ES and DSF. The accumulated Cu binds to lipoylated DLAT, instigating its aggregation and triggering proteotoxic stress. FDX1 reduces Cu^2+^ to Cu^+^ and facilitates the lipoylation of DLAT. Cu inhibits FDX1-regulated synthesis of Fe–S clusters. This inhibition, combined with DLAT aggregation, leads to cell death. GSH and tetrathiomolybdate bind to Cu ions, restoring the level of free Cu ions and subsequently inhibiting cuproptosis

Cuproptosis is thought to interact with elements of the TCA cycle within mitochondria, precipitating oxidative damage to the mitochondrial membrane and protein lipoylation [7, 56] (Fig. 2), a universal post-transcriptional protein modification pathway [55]. Liquid chromatography–mass spectrometry-based metabolomics analysis of cells treated with a Cu ionophore revealed progressive disarray in metabolites associated with the TCA cycle. The obstruction of electron transport chain complexes I and II significantly mitigates Cu-induced cell death [7]. A comprehensive CRISPR/Cas9 knockout screening, coupled with metabolic and biochemical tests, identified several genes associated with cuproptosis (Table 1). These include ferredoxin 1 (*FDX1*), LA synthase (*LIAS*), lipoyl transferase 1 (*LIPT1*), drolipoamide *S*-acetyltransferase (*DLAT*), dihydrolipoamide dehydrogenase (*DLD*), pyruvate dehydrogenase E1 subunit alpha 1 (*PDHA1*), pyruvate dehydrogenase E1 subunit beta (*PDHB*), metal-regulatory transcription factor-1 (*MTF1*), glutaminase (*GLS*), and cyclin-dependent kinase inhibitor 2A (*CDKN2A*) [7]. Among these, the first seven genes positively regulate cuproptosis, while the last three negatively regulate cuproptosis. FDX1 is a reductase that reduces Cu^2+^ to Cu^+^ and serves as the primary regulator of protein lipoylation. Under FDX1’s influence, LIAS attaches the lipoyl moiety to DLAT, a component of pyruvate dehydrogenase implicated in the TCA cycle. Cu^+^ directly engages lipoylated DLAT via a disulfide bond, instigating abnormal DLAT oligomerization and the subsequent inhibition of the TCA cycle [7]. Furthermore, FDX1 can suppress the synthesis of iron–sulfur cluster proteins and destabilize them, ultimately inducing proteotoxic stress and cell death [7].

**Table 1:** Functions of cuproptosis-related genes

Gene	Role in cuproptosis	Subcellular locations	Function description	References
*FDX1*	Reduce Cu^2+^ to Cu^+^; upstream regulatory factors of LA pathway; deletion attenuates cuproptosis	Mitochondrion matrix	Reduce Cu^2+^ to Cu^+^; regulate steroid hormone synthesis as electron transfer intermediates for mitochondrial cytochrome P450 (CYP450)	[7, 57]
*LIAS*	Downstream effector molecules of FDX1; involved in LA pathway	Mitochondrion	Catalyze the conversion of octanoylated domains into lipoylated derivatives	[7, 58–60]
*LIPT1*	Downstream effector of FDX1; involved in LA pathway	Mitochondrion	Catalyze the transfer of a lipoyl group to the lysine residue of targeted enzymes	[7, 58, 60]
*DLAT*	Lipoylation and Cu^+^ binding lead to DLAT oligomerization and further result in cell death	Mitochondrion matrix	Catalyze the breakdown of pyruvate into acetyl-CoA as the E2 subunit of PDC complex	[7, 61]
*DLD*	Not described	Mitochondrion and nucleus	The E3 subunit of PDC complex	[7, 62]
*PDHA1*	Not described	Mitochondrion matrix	Catalyze the conversion of pyruvate to acetyl-CoA as the E1 subunit A1 of PDC complex	[7, 61]
*PDHB*	Not described	Mitochondrion matrix	Catalyze the conversion of pyruvate to acetyl-CoA as the E1 subunit B of PDC complex	[7, 61]
*MTF1*	Deletion results in increased sensitivity to cuproptosis	Cytoplasm and nucleus	Active the transcription of metallothionein and other genes involved in the homeostasis of heavy metals	[7, 63]
*GLS*	Deletion results in increased sensitivity to cuproptosis	Cytoplasm and mitochondrion	Catalyze the breakdown of glutamine into glutamate	[7, 64]
*CDKN2A*	Deletion results in increased sensitivity to cuproptosis	Cytoplasm and nucleus	Arrest the cell cycle in the G1 phase and G2 phase by forming complexes with CDK4/6, and cyclin D	[7, 65]
*ATP7A/B*	Loss of function results in intracellular copper accumulation	Cell membrane, TGN membrane, and plasma membrane	Copper-transporting P-type ATPases	[66–68]
*SLC31A1*	Overactivation results in intracellular copper accumulation	Cell membrane	Promote copper uptake as a high-affinity copper transporter	[69]

While the molecular mechanisms of cuproptosis remain elusive, recent studies have unveiled some potential regulators influencing this process. Liu et al. found that ES-Cu treatment induces an interaction between FDX1 and glucose-6-phosphate dehydrogenase (G6PD), leading to G6PD destabilization [70]. This subsequently reduces nicotinamide adenine dinucleotide phosphate and GSH levels [70], thereby intensifying cuproptosis, suggesting a direct modulation of ROS levels by FDX1 in downstream pathways. In gastric tumors, RNA methyltransferase METTL16 could promote cuproptosis by facilitating FDX1 accumulation through m6A modification on *FDX1* mRNA [71]. This process is inhibited by deacetylase SIRT2 that deacetylates METTL16 at K229 [71]. Additionally, adrenomedullin could reduce FDX1 transcription and suppress cuproptosis in renal cell carcinoma (RCC) through promoting p38/MAPK signaling pathway-mediated phosphorylation and nuclear translocation of Forkhead box O3 (FOXO3) [72]. While FDX1’s role in cuproptosis is critical, there are still unanswered questions about this process that warrant further exploration. First, the precise molecular mechanisms underlying FDX1-regulated DLAT oligomerization and Fe–S degradation remain elusive. Second, the downstream pathways of DLAT oligomers, including the direct connection between DLAT oligomerization, proteotoxic stress, and cell death, remain ambiguous. Undoubtedly, future research will progressively unravel these mysteries.

### Cross-communication of cuproptosis and regulated cell death

Previous studies investigating Cu-induced cell death, it was discovered that cell death induced by ES/Cu and DSF/Cu involves apoptosis [28, 36, 45, 46], and ferroptosis [47–50], suggesting crosstalk between cuproptosis and other forms of cellular death.

Ferroptosis is a form of regulated cell death (RCD) induced by the destruction of iron homeostasis and the accumulation of ROS in lipids. Wang and colleagues recently observed that ferroptosis inducers, sorafenib and erastin, can enhance cuproptosis in primary liver cancer cells, mainly through upregulating FDX1 protein levels and promoting the aggregation of lipoylated proteins [73]. In PDAC cells, Cu can exacerbate erastin-induced ferroptotic cell death by promoting the macroautophagic/autophagic degradation of glutathione peroxidase 4 (GPX4) [74], a protein that blocking ferroptosis by eliminating phospholipid hydroperoxides. These studies suggest that cuproptosis and ferroptosis may have some common triggers or share some signaling pathways. An important intersection between ferroptosis and cuproptosis is mitochondrial metabolism. In cysteine-deprivation-induced ferroptosis, the accumulation of ROS and lipid peroxidation is primarily due to enhanced mitochondrial respiration and rapid depletion of GSH [75]. Excessive accumulation of Cu within cells can also lead to depletion of GSH and generation of a large amount of ROS [76], providing certain conditions for initiating ferroptosis. However, Tsvetkov and colleagues observed that ferrostatin-1, a ferroptosis inhibitor, cannot rescue cells from growth inhibition induced by ES-Cu [7], suggesting the existence of intricate distinguishing mechanisms between cuproptosis and ferroptosis, which is an urgent scientific question in this field.

Cellular damage resulting from Cu overload is also believed to be associated with apoptosis. A major contributor to the cytotoxicity of Cu ions is the generation of ROS via the Fenton reaction. Excessive ROS can trigger apoptosis through multiple mechanisms, including mitochondrial damage, death receptor activation, and exacerbation of endoplasmic reticulum (ER) stress [77]. Previous studies have shown that CuSO_4_ treatment increased ROS levels and decreased GSH levels in cells, which promoted the release of cytochrome c into the cytosol and the activation of caspase-9 and caspase-3, thus activating the mitochondrial apoptotic pathway [78, 79]. Additionally, researchers have observed that administration of CuSO_4_ upregulated the expression of the C/EBP homologous protein (CHOP), Jun N-terminal kinase (JNK), and Caspase-12 in mouse liver cells [80], major features of ER stress-induced apoptosis. As mentioned above, Cu ionophore combined with Cu can induce ROS generation in numerous tumor cells [31–41]. Interestingly, ROS clearance does not always effectively mitigate Cu-mediated cellular damage [7, 32], suggesting that exacerbating ROS production may not be the primary manifestation of Cu cytotoxicity. One possible explanation is that Cu binding to lipoylated components of the TCA cycle is crucial for triggering cellular damage, and its downstream signaling may involve multiple pathways of cell death, such as apoptosis and ferroptosis. Furthermore, different cell types exhibit varying tolerance and response to ROS, leading to diverse pathways of Cu-induced cellular damage in various cells. Further studies are needed to elucidate the crosstalk between cuproptosis and other RCD, which is critical for improving the efficacy of tumor therapy targeting cuproptosis.

## Copper and cancer

**Fig. 3:**
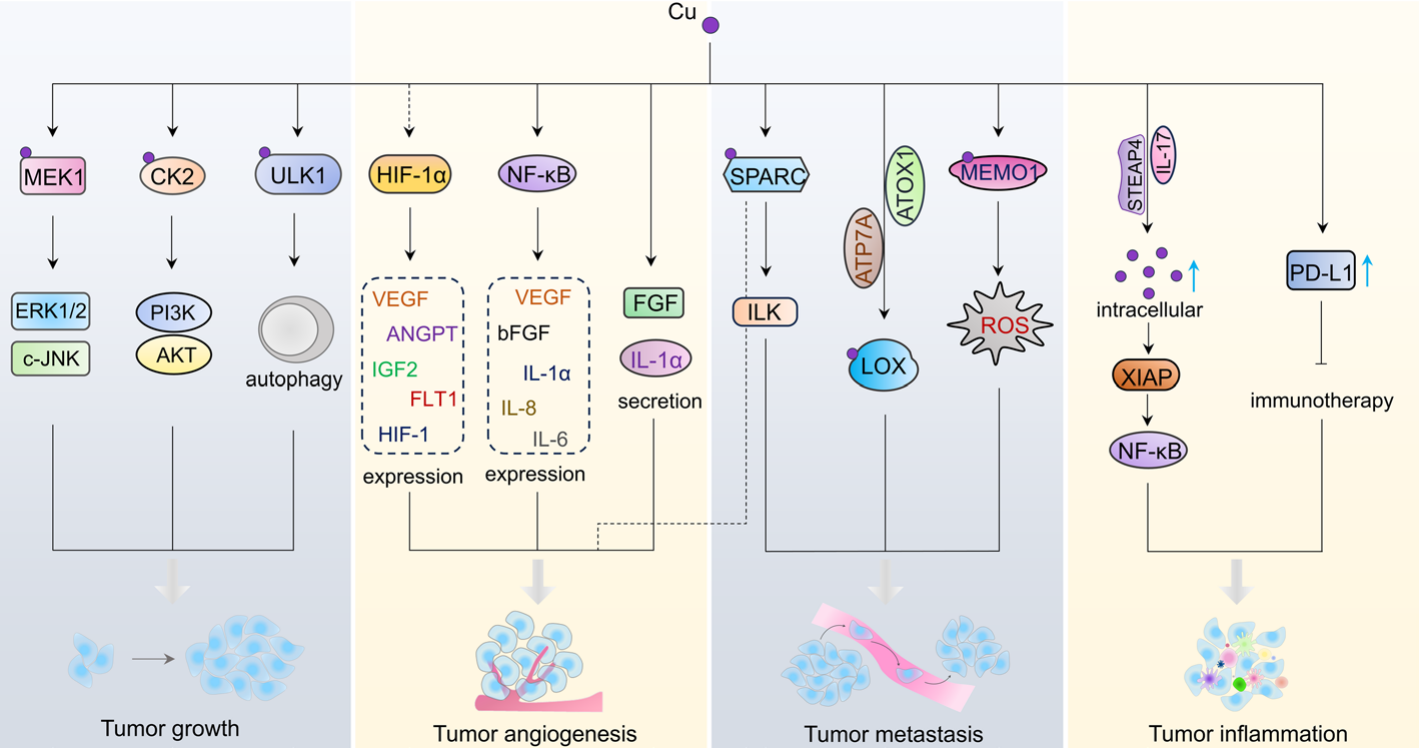
Cu and cancer. Cu directly binds to MEK1, CK2, and ULK1, thereby activating or enhancing signaling pathways associated with tumor growth. Cu activates HIF-1α and NF-κB, thus facilitating the expression of pro-angiogenic factors. Cu promotes the secretion of angiogenic molecules. Cu promotes tumor metastasis by binding to SPARC, LOX, and MEMO1. Cu modulates tumorigenesis-associated chronic inflammation through the IL-17–STEAP4–XIAP axis. Cu also upregulates the expression of PD-L1, consequently inhibiting the efficacy of tumor immunotherapy

The role of Cu in cancer progression has been the subject of significant research owing to its potential contribution to the onset and development of cancer, as it can stimulate cell proliferation [81–83], angiogenesis [9, 84], and metastasis [85–87] (Fig. 3). It has been observed that cancerous cells generally have a greater need for Cu than their healthy counterparts [8]. Elevated levels of serum Cu ions have been reported to be associated with increased risk of cancers, including breast [88–90], lung [91, 92], cervical [93], oral [94], bladder [95], and pancreatic cancer [96]. Moreover, the introduction of Cu sulfate (CuSO_4_) has been found to hasten tumor growth in animal models [97].

### Cu and cuproplasia

Cu plays a critical role in regulating cell growth and proliferation. This Cu-dependent process of cell growth and proliferation is referred to as cuproplasia [8]. Cu primarily regulates various kinase signaling that promotes tumorigenesis by binding and activating key molecules (Fig. 3). For instance, the mitogen-activated extracellular signal-regulated kinase 1 (MEK1) pathway is a Cu-directly regulated signaling pathway. Cu can bind to MEK1, which activates the mitogen-activated protein kinase (MAPK) signaling pathway [98]. This promotes tumor growth through the activation of extracellular signal-regulated kinase 1/2 (ERK1/2) and c-JNK [98–100]. Besides MEK1, Casein Kinase II (CK2) is a Cu-binding protein kinase [101]. As a critical kinase, CK2 regulates various signaling pathways by phosphorylating different substrates, such as the Janus kinase 2 (JAK2)/signal transducer and activator of transcription 3 (STAT3) and NF-κB pathways. In tumor cells, Cu-activated CK2 can promote tumor growth by further activating the phosphoinositide 3-kinase (PI3K)/AKT pathway [101].

### Cu and autophagy

Autophagy can facilitate the cellular recycling of substances and energy. During tumor growth, enhanced autophagy contributes to cancer cell survival by recycling waste and meeting energy demands to some extent [102]. It has been reported that Cu is an important metal ion involved in regulating cellular autophagy. For instance, Cu can activate the Unc-51-like autophagy-activating kinase (ULK) and its associated autophagy pathway via direct interaction with ULK [103]. In animal models of lung cancer driven by KRAS-G12D, deletion of CTR1 diminished the Cu-mediated activation of ULK, which led to a blockage in the autophagic flux, ultimately resulting in tumor growth suppression [103]. Moreover, in lung adenocarcinoma (LUAD) driven by B-Raf proto-oncogene (BRAF), reducing Cu levels inhibited the initiation of ULK-dependent autophagy, thereby bolstering the antitumor efficacy of traditional MAPK pathway inhibitors that induced the upregulation of protective autophagy [104]. The adenosine 5′-monophosphate (AMP)–activated protein kinase (AMPK)–mammalian target of rapamycin (mTOR) pathway serves as a pivotal energy-sensing signal axis within cells. Decreasing of ATP levels can trigger the activation of AMPK, which, in turn, inhibits mTOR, resulting in the upregulation of autophagy, thus promoting the recycling of cellular materials and energy [105]. Liao et al. observed that, in the kidneys of broiler chickens, Cu can induce cellular autophagy by activating the AMPK–mTOR pathway [106], suggesting that Cu may regulate tumor cell autophagy by modulating the AMPK–mTOR signaling pathway. Moreover, it is well known that the activated PI3K/AKT pathway can drive cellular autophagic flux. Given that Cu can activate the PI3K/AKT signaling pathway by binding to CK2 [101], it suggests a potential role for Cu in regulating the autophagy levels of tumor cells via CK2, thereby promoting tumor cell growth. However, intriguingly, Fan Xia et al. found that Cu can directly interact with autophagy-related gene 4B (ATG4B), a crucial regulator in the autophagy process responsible for priming and delipidation of LC3, thereby inhibiting the cysteine protease activity of ATG4B and consequently suppressing cellular autophagy flux [107]. This indicates that Cu’s regulatory effect on autophagy may be bidirectional, with varying impacts across different cancer types. Indeed, in pancreatic cancer cells, blocking SLC31A1-dependent copper absorption exacerbates cellular autophagy, leading to the suppression of tumor cell death [108].

### Cu and tumor angiogenesis

Previous studies have indicated that Cu ions significantly contribute to tumor angiogenesis, a process involving the creation of vascular tubules and new blood vessels, which is vital for tumor growth [109]. McAuslan et al. revealed that Cu can trigger the migration of endothelial cells [110], which is a preliminary stage in angiogenesis. Consequently, silencing *CTR1* can suppress angiogenesis by blocking the entry of Cu into endothelial cells [111]. Furthermore, Cu can stabilize HIF-1α and enhance its binding to crucial sequences within the promoters of its target genes, resulting in increased expression. This includes vascular endothelial growth factor (VEGF), a potent angiogenic factor that fosters tumor angiogenesis [112]. Additionally, Cu can modulate the secretion of angiogenic molecules such as fibroblast growth factor (FGF) and the inflammatory cytokine IL-1α [113, 114]. A deficiency in Cu inhibits the transcriptional activity of NF-κB, consequently reducing the expression of pro-angiogenic factors such as bFGF, VEGF, IL-8, IL-6, and IL-1α [115]. Moreover, the Cu chaperone ATOX1 has been identified as a significant player in angiogenesis. *Atox1* deletion hampered the migration of vascular smooth muscle cells, which is typically driven by platelet-derived growth factor [116]. This deletion also suppressed the infiltration of inflammatory cells that generate angiogenic cytokines, including VEGF and tumor necrosis factor alpha (TNFα) [117]. Collectively, these findings suggest that Cu’s capacity to regulate various angiogenic factors may underpin its role in tumor progression.

### Cu and tumor metastasis

Research indicates that Cu can trigger enzymes and signaling pathways related to metastasis. For instance, the secreted Cu-binding glycoprotein-secreted protein, acidic and cysteine-rich, is a critical regulator of tumor cell invasion and metastasis [118]. Similarly, the cuproenzyme lysyl oxidase (LOX) has been found to promote tumor cell invasion and metastasis (Fig. 3). A study by Shanbhag et al. revealed that a reduction in ATP7A led to decreased LOX activity, consequently suppressing tumor growth and metastasis in a mouse model of breast cancer [87]. The silencing of *Atox1* expression resulted in a decrease in LOX activity and inhibited the migration of breast cancer cells [119, 120], indicating that ATOX1’s role in the ATP7A–LOX signaling pathway is associated with metastasis. Furthermore, Memo was reported to be a Cu-dependent redox enzyme that is required for breast cancer metastasis [86]. This is achieved by increasing ROS concentrations in cellular protrusions and promoting O^2−^ production [76]. Additionally, in triple-negative breast cancer (TNBC) cells, the depletion of Cu selectively targets a distinct subset of highly metastatic SOX2/OCT4^+^ cells for lung metastasis inhibition. Within these SOX2/OCT4^+^ metastatic cells, the suppression of tumor metastasis mediated by Cu depletion relies on the activation of AMPK and the inhibition of mTORC1 [85], suggesting that targeting AMPK–mTORC1 signaling can suppress Cu-induced breast cancer metastasis.

### Cu and tumor immunity

Furthermore, Cu constitutes an indispensable trace element within the body’s immune system. Presently, the molecular mechanisms by which Cu regulates the immune system are not fully elucidated. However, it is unclear whether Cu deficiency could significantly impair the immune system, affect immune cell function, including B cells, T cells, and neutrophils, and inhibit the synthesis or secretion of cytokines like IL-2 [121]. The abnormalities of the immune system function also play pivotal roles in tumorigenesis and tumor therapy. Consequently, Cu may influence the development or therapeutic outcomes of tumors by affecting the immune system. A previous study reported that the inflammatory cytokine IL-17 enhances intratumoral Cu uptake through the activation of STEAP4 [122], a metalloreductase. Elevated levels of Cu lead to the activation of X-linked inhibitors of apoptosis, thereby promoting NF-κB activation and colorectal cancer development [122]. Additionally, Valli et al. found that high levels of Cu in tumor cells upregulate the programmed death-ligand 1 (PD-L1) expression, an immune checkpoint inhibitor linked to cancer immune evasion, at both the mRNA transcription and protein stabilization level [123]. Cu-chelating agents have been demonstrated to promote tumor tissue infiltration by CD8^+^ T and natural killer cells, thereby inhibiting tumor growth [123]. These findings suggest that, from an immunological perspective, Cu may positively regulate tumorigenesis and immunotherapy resistance.

Cu plays a significant role in tumor metabolism, including glycolysis and lipolysis. It has been demonstrated to inhibit tumor growth by reducing the expression of glycolysis-related molecules such as S6 kinase 1 [124] and pyruvate kinase M2 [125]. Moreover, Cu can moderate lipolysis through its interaction with the cysteine residues of phosphodiesterase 3B [126], a molecule responsible for cyclic AMP degradation. Overall, Cu’s capacity to regulate various factors involved in carcinogenesis highlights it as a promising area for continued research. Cu has a critical regulatory function in tumorigenesis and its treatment. Thus, studying Cu’s molecular mechanisms and regulating effects may help us comprehend tumor development and develop efficient diagnostic and therapeutic methods.

## Cuproptosis and cancer

### Cuproptosis-related genes and cancer

Given that certain crucial genes can regulate cuproptosis, comprehending the relationship between these genes and tumors may facilitate the development of cancer treatment strategies hinged on cuproptosis. Numerous studies have shown the significance of cuproptosis-associated genes in tumorigenesis and tumor therapy (Table 2).

**Table 2:** Relationship of cuproptosis-related genes and cancer

Gene	Cancer	Expression	Clinicopathological features	References
*FDX1*	KIRC	Down	Increased expression of *FDX1* was associated with a more favorable OS in patients with KIRC. A lower expression of *FDX1* may heighten the responsiveness to immunotherapies in patients	[127]
	HCC	Down	Higher *FDX1* expression demonstrated prolonged survival in HCC. High *FDX1* levels were correlated with a decrease in PD-1 expression, as well as an enhancement in natural killer cells, macrophages, and B cells within tumor tissues	[128, 129]
	Glioma	Up	*FDX1* acted as a risk factor for OS in glioma	[130]
	Thyroid carcinoma	Down	Higher *FDX1* expression was correlated with the OS in patients with thyroid carcinoma	[131]
	ACC	Down	Higher *FDX1* expression was associated with longer survival in patients with ACC	[132]
	CRC	Down	Low *FDX1* expression was associated with poorer OS in patients with CRC	[133]
*LIAS*	Glioma	Up	High *LIAS* expression was associated with poorer OS in patients with glioma	[134]
	Lung cancer	Up	High *LIAS* expression was associated with poorer OS in patients with lung cancer	[135]
	KIRC	Down	High *LIAS* expression was linked with good OS and DSF in patients with KIRC	[135]
	READ	Down	High *LIAS* expression displayed good OS in patients with READ	[135]
	Ovarian cancer	Down	High *LIAS* expression displayed good OS and PFS in patients with ovarian cancer	[135]
	Breast cancer	Down	High *LIAS* expression was linked with good OS in patients with breast cancer	[135]
*LIPT1*	Glioma	Up	*LIPT1* was a risk factor for OS in patients with glioma	[130]
	LIHC	Up	Low *LIPT1* expression was associated with significantly longer OS in patients with LIHC	[136]
	HCC	Up	High *LIPT1* expression was associated with more malignant pathological features and poor prognosis in patients with HCC	[137]
	NSCLC	Down	High *LIPT1* expression was associated with a favorable prognosis for patients with NSCLC	[138]
	PAAD	Down	*LIPT1* acted as a protective factor for OS in patients with PAAD. Silencing *LIPT1* promotes the proliferation, migration, and invasion of PANC-1 and SW1990 cells	[139]
*DLAT*	Glioma	Up	High *DLAT* expression was associated with poorer survival in patients with glioma	[134]
	ESCA	Up	*DLAT* acted as a risk factor for OS in patients with ESCA	[140]
	BRCA	Down	*DLAT* acted as a risk factor for OS in patients with BRCA	[140]
	COAD	Down	*DLAT* served as a protective factor for OS in patients with COAD	[140]
	READ	Down	*DLAT* served as a protective factor for OS in patients with READ	[140]
	KIPAN	Down	*DLAT* served as a protective factor for OS in patients with KIPAN	[140]
	KIRP	Down	*DLAT* served as a protective factor for OS in patients with KIRP	[140]
	LIHC	Up	High expression levels of *DLAT* were linked with poor OS in patients with LIHC. Elevated levels of *DLAT* were identified in patients exhibiting resistance to 5-fluorouracil and lenvatinib. Higher levels of *DLAT* expression were correlated with elevated PD-L1 levels	[140–142]
	PAAD	Up	Elevated *DLAT* expression in PAAD was correlated with increased resistance to a range of chemotherapeutics, including gemcitabine, irinotecan, 5-fluorouracil, and oxaliplatin	[143]
	KIRC	Down	High expression levels of *DLAT* were linked with good OS in patients with KIRC	[140, 142, 144]
	LUAD	Up	High expression levels of *DLAT* were associated with worse outcomes in patients with LUAD	[145]
*GLS*	PCA	Up	High expression of *GLS* was significantly associated with Gleason score and tumor stage	[146]
*CDNK2A*	Pan-cancer		*CDKN2A* mutations that prevent its binding to CDKs can lead to uncontrolled growth in various tumors, including NSCLC, PAAD, HNSCC, breast, and ovarian cancers. Epigenetic reduction of CDKN2A level changed the expression of cancer-related oncogenes or tumor suppressor genes and promoted tumorigenesis	[147]
*MTF1*	Gastric cancer	Down	High *MTF1* expression was associated with longer OS in patients with gastric cancer	[148]
	LIHC	Up	High *MTF1* expression was related to poor prognosis in patients with LIHC	[149, 150]
	LGG	Up	High *MTF1* expression was related to poor prognosis in patients with LGG	[149]
	KIRC	Down	High *MTF1* expression was associated with a good prognosis in patients with KIRC	[149]
	Lung cancer	Down	High *MTF1* expression was associated with good prognosis in patients with lung cancer	[149]
	Ovarian cancer	Down	High *MTF1* expression was associated with good prognosis in patients with ovarian cancer	[149]
	Breast cancer	Down	High *MTF1* expression was associated with good prognosis in patients with breast cancer	[149]
*SLC31A1*	Glioma	Up	*SLC31A1* was a risk factor for OS in patients with glioma	[130]
	Breast cancer	Up	High *SLC31A1* expression was correlated with a poorer prognosis in patients with breast cancer	[151–153]
	BRCA	Up	Higher *SLC31A1* expression was associated with worse OS in patients with BRCA	[154]
	STAD	Up	Lower *SLC31A1* expression was associated with worse OS and DSF in patients with STAD	[154]
	KIRC	Down	Lower *SLC31A1* expression was associated with worse OS and DSF in patients with KIRC	[154]
	LGG	Up	Low *SLC31A1* expression was associated with good OS in patients with LGG	[155]
	Bladder cancer	Up	Higher *SLC31A1* expression was associated with worse OS in patients with bladder cancer	[156]
*PDHA1*	Glioma	Down	*PDHA1* was a favorable factor for prognosis in patients with glioma	[130]
	HCC	Up	High *PDHA1* expression was associated with a poor survival rate in patients with HCC	[157]
	OSCC	Down	Low *PDHA1* expression was correlated with good survival in patients with OSCC	[158]
*ATP7A*	Glioma	Up	*ATP7A* were risk factors for OS in patients with glioma	[130]
	HCC	Up	High ATP7A expression was correlated with a poorer prognosis in patients with HCC. Low expression of ATP7A increased HCC cell copper accumulation and suppressed tumor cell growth	[159]
	OSCC	Down	Low *ATP7A* expression was correlated with good survival in patients with OSCC	[158]
*ATP7B*	Glioma	Up	*ATP7B* were favorable factors for OS in patients with glioma	[130]

#### *FDX1*

FDX1, a pivotal modulator of cuproptosis, is vital for various metabolic processes. A strong correlation has been detected between FDX1 and cancer. A notable shift in glucose, amino acid, and fatty acid oxidative metabolism has been observed in LUAD following FDX1 downregulation [160]. *FDX1*’s underexpression in kidney renal clear-cell carcinoma (KIRC), confirmed at protein and mRNA levels, contrasts with a more favorable overall survival prognosis when its expression is increased [127]. The study also proposed that *FDX1* lower expression may increase sensitivity to immunotherapies in patients [127]. Zhang et al. identified an *FDX1* downregulation in HCC cells, while patients with higher *FDX1* expression exhibited extended survival [128]. This finding was echoed by Quan et al., who observed a significant enhancement of survival among liver patients with HCC with elevated FDX1 expression. Furthermore, FDX1 knockdown stimulated the proliferation and migration of hepatic cancer cells by reducing cuproptosis [129]. They also reported a significant enhancement in natural killer cells, macrophages, and B cells in tumor tissues with higher *FDX1* expression. Programmed death-1 (PD-1) expression was low in these tissues, suggesting a potential regulatory role of FDX1 in immune infiltration in HCC [129]. Contrarily, in glioma, *FDX1* expression levels were upregulated compared with normal tissues. FDX1 knockdown notably hindered aerobic glycolysis and glioma cell proliferation [134]. Li et al. further highlighted that the *FDX1* expression is promoted by the c-Myc-YTHDF1 signaling pathway in glioma cells and demonstrated that the c-Myc-YTHDF1/FDX1 axis inhibited the mitophagy and promoted the malignant phenotype of glioma cells [161]. In summary, these studies depict a close connection between FDX1 and clinical characteristics, anticancer drug sensitivity, immune-related pathways, and immune cell infiltration in various cancer types. This underlines the potential of FDX1 as a future target for cancer treatment and a prognostic biomarker.

#### ***LIAS***, ***LIPT1***

LA, a mitochondrial antioxidant, is vital for eliminating free radicals and regulating mitochondrial energy metabolism and oxidative stress [162]. The proteins expressed by *LIPT1* and *LIAS* are integral parts of the LA pathway, which facilitates the post-transcriptional lipoic modification of proteins such as the pyruvate dehydrogenase complex (PDC) [58–60]. This pathway is fundamental for standard cellular activity and cuproptosis. LIAS, an enzyme involved in LA metabolism, catalyzes the final phase of LA synthesis, while LIPT1 facilitates the transfer of a lipoyl group to the lysine residue of targeted enzymes [58–60]. *LIAS* expression levels are often linked to the prognostic outcomes in various patients. For instance, elevated *LIAS* expression is generally associated with a poorer prognosis in lung cancer. Concurrently, it tends to indicate a more favorable prognosis in KIRC, rectum adenocarcinoma (READ), ovarian cancer, and breast cancer [135]. Furthermore, *LIPT1* has been associated with tumor progression. For example, in gliomas and liver cancer, higher levels of *LIPT1* expression were indicative of a poorer survival rate [130, 136, 137].

#### *DLAT*

DLAT, the E2 subunit of the PDC complex, is vital for the TCA cycle. Its lipoylation by LIAS, instigated by the presence of Cu, leads to oligomerization and subsequently induces cell death [7]. Several studies have highlighted the role of DLAT in tumor formation. Using an array of bioinformatics tools, Xu et al. revealed that *DLAT* exhibits abnormal expression patterns in most malignant tumors. They unveiled a correlation between DLAT and various immunogenes, including the major histocompatibility complex, immune stimulators and inhibitors, and chemokines and their receptors [140]. Furthermore, *DLAT* expression was linked to the tumor microenvironment (TME), along with a diverse infiltration of immune cells [140]. These findings emphasize DLAT’s importance in cancer development and immunity. In the context of non-small-cell lung cancer (NSCLC), it was found that PM2.5 could escalate *DLAT* expression, thereby fueling glycolysis and amplifying tumor cell proliferation. This process is believed to be related to the dual regulatory mechanism of the Sp1–DLAT and eIF4E–DLAT axis [163]. In HCC, *DLAT* expression was higher than in normal tissues, correlating with more severe clinical features [141, 142]. Higher *DLAT* expression was linked to elevated PD-L1 levels in HCC [141]. Furthermore, in contrast to normal tissues, *DLAT* upregulation coincided with an increase in Treg cells in HCC [142]. In patients exhibiting resistance to 5-fluorouracil and lenvatinib, elevated DLAT levels were observed, suggesting a potential role for DLAT in fostering drug resistance [142]. A similar trend was observed in pancreatic adenocarcinoma (PAAD), with elevated *DLAT* expression correlating with increased resistance to a range of chemotherapeutics, including gemcitabine, irinotecan, 5-fluorouracil, and oxaliplatin [143]. These studies suggest that DLAT may be a critical target for liver cancer immunotherapy. Conversely, DLAT was identified as a tumor suppressor in KIRC [144]. DLAT overexpression inhibited cell growth, migration, and invasion of KIRC cell lines [164].

In summary, these investigations suggest DLAT as a pivotal regulator in tumorigenesis and tumor treatment, positioning it as a promising biomarker and potential therapeutic target. However, the regulatory functions of DLAT may vary across different tumors. Further explorations of the molecular mechanisms involving DLAT could enhance our understanding of its role in tumors. Moreover, in cuproptosis, DLAT primarily induces cell death through lipoylation and oligomerization. Hence, delving deeper into the molecular mechanisms of DLAT’s lipoylation and oligomerization in tumor cells could shed light on the relationship between cuproptosis, tumorigenesis, and tumor treatment.

#### *GLS*

GLS primarily facilitates the breakdown of glutamine into glutamate, a process integral to the Krebs cycle [64]. This cycle generates ATP and produces various amino acids, lipids, and nucleotides [165]. Recent studies reveal that GLS is vital for the emergence and progression of various solid cancers. Reduction in *GLS* expression is associated with significant suppression of cell growth and proliferation in glioblastoma [166], prostate cancer (PCA) [146], and melanoma [167]. Overexpressed synaptosomal-associated protein 25 (SNAP25) has been demonstrated to regulate GLS-mediated glutaminolysis, thereby inhibiting glioma cell growth in vitro and in vivo [168]. This suggests that SNAP25 could potentially act as an upstream regulator of GLS. Furthermore, glioma cell growth inhibition through *GLS* silencing is believed to be linked with c-myc, Bid, and Bcl-2 mediated apoptosis [166]. Moreover, GLS is implicated in radioresistance. In PCA and stem cells, the high demand for glutamine enhances the radioresistance of cells. This correlates with high expression levels of GLS and MYC, two key regulators of glutamine metabolism, and is significantly associated with reduced progression-free survival (PFS) in patients with PCA undergoing radiotherapy [169]. Considering that GLS knockout enhances cuproptosis sensitivity [7], targeting GLS could be a beneficial strategy to counteract the drug or radioresistance of tumor cells.

#### *CDKN2A*

CDKN2A, also known as p16CDKN2A/p16INK4, is a 16 kD cell-cycle inhibitor protein that inhibits the cell cycle by forming complexes with cyclin-dependent kinases (CDK) 4 and 6 and cyclin D. This action impedes the kinase activity of the enzyme and results in the arrest of the cell cycle in the G1 and G2 phases [65, 147]. Historically, research has primarily centered on CDKN2A’s role as a tumor suppressor [147]. CDKN2A mutations that prevent its binding to CDKs can lead to uncontrolled growth in various tumors, including NSCLC, PAAD, head and neck squamous cell carcinoma (HNSCC), and breast and ovarian cancers [147]. Furthermore, its expression level has proven significant in the growth and treatment of these tumors. Hypermethylation of *CDKN2A* promoter is a leading cause of reduced CDKN2A expression in tumor cells. An increasing body of evidence suggests that CDKN2A’s epigenetic aberration relates to epigenetic changes in cancer-related oncogenes or tumor suppressor genes [147]. For instance, *CDKN2A* promoter methylation in LUAD was associated with KRAS and EGFR mutations [170]. Although Tsvetkov et al. observed that CDKN2A knockout could increase the sensitivity to cuproptosis [7], indicating that CDKN2A is an anti-cuproptotic protein, the specific molecular mechanisms remain unclear. Further exploration will shed light on the effective targets involved in CDKN2A-regulated cuproptosis and enhance tumor therapy.

#### *MTF1*

MTF-1, a transcription factor, is instrumental in activating the transcription of the Cu-binding protein metallothionein (MT) [63]. Acting as a Cu reservoir, MT helps maintain metal homeostasis and guard against injury precipitated by metal overload [171]. In gastric cancer, *MTF1* expression is relatively subdued, which correlates with a dire prognosis [148]. Paradoxically, *MTF1* expression is increased in HCC cells, resulting in poor survival rates and recurrence [150]. The MTF1 overexpression can spur the proliferation and metastatic potential of HCC cells [150]. Recent studies by Song et al. substantiate the theory that MTF1 knockdown curbs cell proliferation, escalates ROS, and exacerbates cell death in HepG2 and Huh7 [149]. Furthermore, they found that high *MTF1* expression was linked to a poor prognosis for liver HCC and brain lower-grade glioma (LGG) but a better prognosis for KIRC, lung, ovarian, and breast cancer [126]. Single-cell sequencing revealed that MTF1 was implicated in several tumor development-associated processes, including DNA repair, angiogenesis, and cell invasion [149]. Zhang et al. found that zinc could elevate MTF1 expression and activate ERK1/2 and AKT signaling pathways in ovarian cancer cells [172]. The impact of zinc on tumorigenesis varies substantially across different types of tumor cells. For example, elevated zinc concentrations can benefit ovarian cancer cells by promoting migration, invasion, and epithelial-to-mesenchymal transition [172] while concurrently inhibiting PCA cell growth [173, 174]. Considering that MTF1 can reduce a cell’s sensitivity to cuproptosis [7], it is plausible that higher zinc concentrations could protect tumor cells from cuproptosis. This suggests that zinc negatively modulates cuproptosis, implying that cells with lower zinc levels could exhibit greater sensitivity to cuproptosis. Moreover, a recent study reported that LATS, a kinase of the Hippo pathway that governs organ size and cancer development, could disrupt heavy metal homeostasis and reduce cellular protection by phosphorylating and inhibiting MTF1 [175], suggesting a possible role for LATS in cuproptosis. Collectively, these findings indicate that MTF1 could be a crucial factor in the progression of various human cancers. Because MTF1 knockout could enhance cell sensitivity to cuproptosis, targeting MTF1 and its associated signaling pathway could be a potential treatment strategy for cancer.

#### *SLC31A1*

SLC31A1, also known as CTR1, is a high-affinity Cu transporter. It is organized as a homotrimer on the cell membrane, assisting in the inflow of Cu. It has been reported that the suppression of SLC31A1 expression could inhibit tumor growth propelled by the BRAF/MEK/ERK signaling pathway [98, 99], a classic characteristic of several malignant tumors. Furthermore, a feedback loop appears to exist between MEK and SLC31A1 to modulate tumorigenesis. Jin et al. observed in pancreatic ductal adenocarcinoma cells that the MEK signal pathway sustains the high expression level of SLC31A1 by curbing the expression level of miR-124, which can suppress the SLC31A1 expression by binding *SLC31A1* 3′ UTR [176]. This suggests that the MEK signaling pathway could potentially be targeted to alter Cu absorption by tumor cells. Through the analysis of websites and datasets, Kong and colleagues observed that *SLC31A1* expression is elevated in most tumor types, including adrenocortical carcinoma, mesothelioma, and LGG, compared with nontumor tissues. This increased expression was shown to correlate with shorter overall and disease-free survival [154]. Furthermore, *SLC31A1* expression levels are positively associated with immune cell infiltration [154]. This observation was also replicated in breast cancer samples, where *SLC31A1* upregulation predicted a dismal prognosis and was associated with a suppressed immune response and metabolic pathways [151, 152]. Taken together, these findings suggest that SLC31A1 could present a new target for adjusting Cu balance or cuproptosis in tumor cells.

#### *ATP7A*/*ATP7B*

ATP7A and ATP7B are analogous isoforms of Cu-transporting P-type ATPases. Mutations in *ATP7A* and *ATP7B* can lead to neurodegeneration, causing Menkes and Wilson’s disease, respectively [67, 68]. In breast cancer, ATP7A suppression can impede the function of LOX, a modulator of extracellular matrix (ECM) remodeling, which can consequently lead to tumor cell migration [85, 120]. Besides Cu transport, ATP7A and ATP7B can interact with platinum drugs and pump them across membranes, contributing to the resistance of tumor cells to platinum-based cancer treatments. In patients with NSCLC, those testing positive for ATP7A were found to have a significantly lower histological grade and reduced response to platinum-based chemotherapy than those testing negative for ATP7A [177, 178]. Comparable results were observed in in vitro cell cultures, where the *ATP7A* silencing could reverse cisplatin resistance and enhance cell apoptosis [179]. ATP7B overexpression has also been linked to less favorable clinical outcomes in patients with esophageal carcinoma undergoing cisplatin-based chemotherapy [178]. These findings suggest that ATP7A and ATP7B expression could potentially serve as markers of cisplatin-related drug resistance in patients with tumors, providing valuable insights for cancer treatment strategies.

#### Scores based on cuproptosis-related genes

Based on the transcriptomic data and matching clinical information on cancer from publicly accessible sources, researchers screened the cuproptosis-related genes using mainstream machine learning algorithms, including least absolute shrinkage and selection operator, gradient-boosted decision trees, decision tree, and Gaussian mixture model, and established the cuproptosis-related gene (CRG) risk score signature as an evaluative measure to access the relationship between cuproptosis and various aspects of tumorigenesis, including initiation, progression, prognosis, and immune infiltration. Huang and colleagues developed a cuproptosis-related gene index (CRGI), integrating ten cuproptosis-associated genes with six biomarkers in PAAD. Their findings revealed a significant survival edge for the group with a lower CRGI. Their experiments identified *DLAT*, *LIPT1*, and *LIAS* within CRGI genes as credible biomarkers of PAAD [180]. Zhang and his team observed that cells within the high cuproptosis-related risk score group that were more reliant on glycolysis for energy exhibited a decrease in FDX1 and an increase in CDKN2, suggesting this group had a resistance to cuproptosis [128]. Conversely, in multiple myeloma, the high-risk group was identified to have elevated levels of β2-microglobulin and lactate dehydrogenase (LDH) and advanced Revised International Staging System (R-ISS) or ISS stages, alongside a higher likelihood of cytogenetic abnormalities [181]. The low-risk group displayed a greater abundance of immune cells and, correspondingly, an increased sensitivity to immunotherapy [181]. A positive correlation was reported between the risk score and stemness index, a measure used to evaluate cancer stem cell activation, a crucial driving component of tumor metastasis, recurrence, progression, and drug resistance [181]. Yao et al. formulated a cuproptosis-related immune risk score (CRIRS) based on PRLR, DES, and LECT2 markers. Their system identified that, in prostate adenocarcinoma, a high CRIRS was indicative of poor overall survival rates, higher T stage, and Gleason scores, which are universally recognized clinicopathological indicators for determining the prognosis of prostate cancer [182]. Furthermore, they uncovered an inverse relationship between high CRIRS and the levels of some immune checkpoints, a type of immunosuppressive molecule expressed on immune cells, and the prevalence of activated immune cells. Lower CRIRS, contrarily, suggested a more favorable response to chemotherapy/targeted drugs and immunotherapy [182]. Moreover, several studies established the CRG risk score signature based on cuproptosis-associated gene expression and confirmed that the CRG risk score is a strong prognostic marker and could contribute to guiding more effective treatment regimens [183–186]. Compared with a single gene expression level, this risk score-associated assessment based on machine deep learning is more accurate in reflecting the role of cuproptosis in tumorigenesis and treatment because cuproptosis is a multiple-gene-regulated biological process. Despite the suggestion of these bioinformatics data analyses that cuproptosis is intricately linked with tumor progression and therapeutic outcomes, these findings still require extensive experimental validation. Additionally, the role of cuproptosis varies in different cancer types, which could be attributed to the varying dependency of various cancer cells on Cu or the diverse ways in which Cu ions regulate cellular metabolism and behavior. Consequently, future research should focus on unraveling the molecular mechanisms of Cu ions and cuproptosis on cancer cells to enable the precise application of cuproptosis in cancer treatment.

### Cuproptosis and tumor treatment

#### Cu ionophores

Cu ionophores can transport Cu across the plasma or mitochondrial membranes. This can subsequently lead to an increase in intracellular Cu levels, potentially inducing cuproptosis. Thus, Cu ionophores could serve as potential therapeutic agents for cancer treatment. Besides ES and DSF mentioned above, various Cu ionophores have been found, including bis(thiosemicarbazone) analogs [187], diacetyl-bis(*N*^4^-methyl thiosemicarbazide) [188], and glyoxal-bis(*N*^4^-methyl thiosemicarbazide) [188]. Among these Cu ionophores, ES and DSF have been demonstrated to elevate intracellular Cu concentrations and display anticancer properties effectively.

##### Elesclomol

ES, a chemotherapeutic adjuvant, was initially developed by Synta Pharmaceuticals for metastatic melanoma treatment. Further clinical trials, while not proving it to be clinically beneficial for patients, provided a comprehensive evaluation of ES safety [6]. A phase III clinical trial indicated that ES, when combined with paclitaxel, did not significantly enhance the PFS of patients with melanoma. However, this study discovered that combination therapy improved PFS in patients with normal LDH levels [189]. Increased serum LDH levels may, in part, reflect increased hypoxia and glycolysis, suggesting that cell energy metabolism may be a crucial factor for assessing ES’s anticancer efficacy of ES. Moreover, while ES has undergone clinical trials for solid tumors, there have been no reports of significant tumor treatment success [6].

Given that Cu ionophore-induced cuproptosis is linked to mitochondrial metabolism, ES has potential as a countermeasure against the drug resistance observed in tumor cells with elevated levels of mitochondrial metabolism. Tumor cells’ robust proliferation and growth hinge on extensive protein synthesis, often leading to an overproduction of misfolded proteins [190]. Accordingly, the degradation of these misfolded proteins via a proteasome-dependent mechanism is critical for tumor cells to avert proteotoxic events. This makes proteasome targeting a viable strategy to curtail tumor cell growth. However, proteasome inhibitors (PI) can significantly impede the growth of most solid tumor cells in vitro. In vivo studies have demonstrated the formidable adaptability of tumors to these inhibitors, which restricts their clinical applicability [191]. Therefore, targeting metabolic reshaping caused by PI can enhance its antitumor capability. Tsvetkov et al. found that an upsurge in mitochondrial metabolism is a key characteristic of proteasome inhibitor-resistant cells. Simultaneously, their research indicated that these PI-resistant cells are more susceptible to ES [192]. Importantly, ES can enhance the sensitivity of PI-resistant cells to PI, suggesting its potential as an anticancer drug designed to overcome PI resistance. Targeted drugs in tumor therapy may also induce amplified mitochondrial metabolism. It has been found that mitochondrial metabolism is enhanced in melanoma cells resistant to the BRAF^V600E^ gene mutation-targeted drug, vemurafenib, and these resistant cells exhibit increased sensitivity to ES [193]. These studies suggest that inducing cuproptosis may overcome drug resistance caused by increased mitochondrial metabolism during cancer treatment.

Besides mitochondrial metabolism, other molecular pathways have been found to play a role in ES-induced cuproptosis. Recent studies by Wen et al. demonstrated that ES-CuCl_2_ could enhance docetaxel-induced mortality in PCA cells in vitro and in vivo [194]. Mechanistically, they revealed that cuproptosis triggered by ES-CuCl_2_ increases the expression level of DLAT, which in turn suppresses cell autophagy and proliferation via the mTOR signaling pathway [168]. This results in augmented sensitivity of tumor cells to docetaxel. Gαq and Gα11, subunits of a heterotrimeric G protein encoded by *GNAQ* and *GNA11*, respectively, are involved in regulating G protein-coupled receptor-related signal transfer. *GNAQ* and *GNA11* mutations are hallmarks of uveal melanoma. These mutations cause continuous activation of Gαq and Gα11, thereby enhancing tumor signaling pathways such as MAPK, PKC, and YAP/TAZ [195]. Li and colleagues observed that ES can selectively hinder the proliferation of uveal melanoma cells harboring *GNAQ/11* mutations through Cu binding. They found that, in these mutant cells, ES could stimulate LATS1, a protein kinase in the Hippo pathway, by producing abundant ROS [196]. Moreover, they noted that ES could counteract the resistance of these mutant cells to the MEK inhibitor binimetinib [170].

Considering that Cu can bind to and activate MEK1, the ability of ES-CuCl_2_ to enhance the effectiveness of MEK inhibitors may depend on intracellular Cu ion distribution, like within the mitochondria. Further investigation into the precise molecular mechanism could offer valuable insights into the relationship between cuproptosis and the MAPK signaling pathway. However, it should be noted that cuproptosis is not the only form of ES-CuCl_2_-induced tumor cell death. Other modes of cell growth inhibition or cell death, such as autophagy and ROS-induced apoptosis, are also involved. Consequently, future research should strive for a more comprehensive understanding of how ES-CuCl_2_ interacts with other forms of cell damage. This will aid in developing more effective cancer treatment strategies.

##### Disulfiram

DSF, an aldehyde dehydrogenase inhibitor, has been used clinically to treat alcohol dependence, also functions as a Cu ionophore, and causes cell death in cancer cells [197]. It has shown great potential as an anticancer agent because of its safety and few side effects. DSF has been found to interact with Cu ions to form bis-diethyldithiocarbamate-Cu (CuET), which may increase ROS levels and contribute to the treatment of various malignant tumors [197]. DSF-Cu can also enhance the drug sensitivity of cancer stem cells by interacting with NPL4, potentially disrupting the p97–NPL4–UFD1 pathway and inhibiting p97’s ubiquitinated protein degradation function, thus leading to cell death [198–200]. However, the exact molecular mechanism by which p97–NPL4–UFD1 regulates cuproptosis remains elusive.

#### Small compounds

Recently, researchers have identified several small molecule compounds that showed the potential to disrupt copper homeostasis and induce or exacerbate cuproptosis, offering promising avenues for cancer therapy. For example, sorafenib, the first multi-tyrosine kinase inhibitor approved for diverse cancers and known to induce ferroptosis, has been found to enhance cuproptosis by increasing FDX1 aggregation and GSH depletion [73]. Zinc pyrithione (ZnPT) was showed to induce cuproptosis in TNBC cells by perturbing intracellular copper homeostasis and promoting DLAT oligomerization [201]. Additionally, the protein synthesis inhibitor anisomycin also was found to induce cuproptosis in tumor cells, likely via inhibiting Yin Yang 1 (YY1)-mediated transcriptional activation of the key genes in the LA pathway (i.e., FDX1, DLD, DLAT, and PDHB) [202]. Moreover, researchers have unveiled that 4-OI could inhibit glycolysis by targeting the GAPDH enzyme to promote ES-Cu-mediated cuproptosis [203].

#### Nanoparticle

The concentration of Cu ions within the cellular environment is critical for inducing cuproptosis in cancer cells. However, the challenge lies in the relatively short blood half-life of small-molecule-based Cu ionophores, which restricts the efficient delivery of Cu to tumor cells. Certain Cu ionophores like ES and DSF have been shown to have a basic safety profile. However, their long-term use can disrupt the balance of other metals in the body, thus potentially amplifying side effects in patients undergoing treatment. Consequently, an approach that specifically targets tumor cells for Cu therapy could provide more accurate cancer treatment. Potential solutions may lie in developing nano-drug delivery systems that release Cu or ionophores directly at the tumor site, thus inducing cuproptosis and enhancing the effectiveness of therapy [204].

Xu et al. designed a nonporous Cu(I) 1,2,4-triazolate(Cu(tz)) coordination polymer nanoplatform engineered with glucose oxidase (GOx), termed GOx@(Cu(tz)), and found this system can effectively stimulate cuproptosis. Upon exposure to GSH stimulation in tumor cells, the platform’s GOx catalytic activity is effectively activated, leading to glucose depletion [205]. Glucose depletion can result in an increase in intracellular H_2_O_2_, which could increase ROS and promote the cell death induced by Cu. The specific environment of tumor cells and tissues is a crucial factor for targeted delivery. Wu et al. developed PEGylated mesoporous silica nanoparticles infused with Cu and DSF. These nanoparticles rapidly degrade, releasing Cu and DSF under the acidic conditions of the tumor environment while remaining stable at physiological pH [206]. Animal experiments demonstrated that this nanosystem effectively curbed the growth of 4T1 cell-derived tumors in female BALB/C nude mice [206]. Furthermore, researchers also tried to co-deliver chemotherapeutic drugs and cuproptosis inducers to tumor tissues to enhance the antitumor efficacy of nanomedicines. For instance, in E-C@DOX NPs, in addition to containing Cu^2+^, they also incorporate the front-line chemotherapeutic drug doxorubicin (DOX) [207]. Within tumor tissues, E-C@DOX NPs not only induce tumor cell cuproptosis but also inhibit the signaling pathways associated with tumor cell stemness and survival, enhance mitochondrial damage, thereby suppressing ATP-dependent drug efflux pathways and reversing DOX resistance in breast cancer [207]. LDH/HA/5-FU nanosheets can specifically target tumor cells and release Cu^2+^ and 5-FU, thereby inducing cuproptosis and apoptosis in tumor cells, exhibiting outstanding inhibitory effects on tumors [208].

Photothermal therapy (PTT) is a noninvasive approach to cancer treatment where photothermal agents use external sources of light energy, especially near-infrared radiation (NIR), to convert it into thermal energy for targeting and destroying tumor cells [209]. Cu possesses localized surface plasmon resonance (LSPR) characteristics, making Cu-based nanomaterials highly effective in NIR absorption and demonstrating outstanding photothermal performance [210]. Ning et al. designed a PV-coated Cu oxide nanoparticle (Cu_2_O)/TBP-2 cuproptosis sensitization system (PTC) by physically extruding Cu_2_O, platelet vesicle, and aggregation-induced luminescence, photosensitizer (TBP-2) [211]. PTC releases Cu ions in tumor cells due to acidic conditions and hydrogen peroxides. Additionally, light irradiation accelerates TBP-2’s entry into the cell membrane to produce hydroxyl radicals, which deplete GSH and restrict the outflow of Cu ions, ultimately causing cuproptosis [211]. Zhou et al. created a photothermally activated nano platform (Au@MSN-Cu/PEG/DSF) and found that it efficiently kills tumor cells and suppresses tumor growth when synergized with PTT [212]. Recently, Zhang et al. established O_2_-PFH@CHPI NPs [213]. Upon NIR, O_2_-PFH@CHPI NPs simultaneously triggered O_2_ release for photodynamic therapy (PDT) to promote oxidative stress, and effectively induced Cu^+^-mediated cuproptosis in HCC cells. Moreover, the tilt of redox balance promoted lipid peroxidation and GPX4 inactivation, resulting in an augmented ferroptosis [213]. These results suggest that combining nanomedicines-based cuproptosis inducer with PTT shows potential in improving the precision and therapeutic effectiveness of tumor treatment.

Besides escalating the Cu toxicity in cancer cells, Cu delivery systems have been employed to craft potent tumor immunotherapy sensitizers. The Xin group devised a nanoparticle (NP@ESCu) that co-encapsulates ES and Cu, exhibiting sensitivity to ROS [214]. The high levels of ROS in cancer cells trigger the release of ES and Cu from nanoparticles, leading to cell death. When applied in vivo, NP@ESCu instigates cuproptosis in conjunction with anti-PD-L1 antibody (αPD-L1), effectively enhancing subcutaneous bladder cancer therapy [214]. The Huang group pioneered a more intricate and thorough cuproptosis-related delivery system (CS/MTO-Cu@AMI) to prime tumor chemoimmunotherapy. In this system, mitoxantrone (MTO) and Cu^2+^ are structured into a nano-metal–organic framework (MTO-Cu) through rational coordination, granting the nanoparticles a pH/GSH dual-responsive release behavior [215]. The nanoparticles underwent further modification with chondroitin sulfate (CS), facilitating tumor targeting and bestowing hyaluronidase-responsive charge-reversal characteristics. Additionally, the inclusion of amiloride (AMI), known for its inhibitory effects on macropinocytosis and exosome secretion, further enhanced the system’s functionality [215]. Upon application, CS/MTO-Cu@AMI activated the AMPK pathway by inducing cuproptosis and mitochondrial dysfunction, thereby orchestrating the PD-L1 degradation. Concurrently, the treatment-induced dsDNA damage in cells stimulated antitumor immunity via the activation of the cGAS–STING pathway [215]. Beyond this, researchers have developed other nano-drug delivery systems to augment tumor cell immunotherapy, such as the CaO_2_@Cu-SS/JQ-1@DSPE-PEG-FA (CCJD-FA) for colorectal cancer (CRC) immunotherapy [216], BSO-CAT@MOF-199 @DDM (BCMD) for glioblastoma immunotherapy [217], Cu-doped BiSex (CBS) for PCA immunotherapy [218], CLDCu for melanoma immunotherapy [219], and PEG@Cu_2_O-ES for breast cancer immunotherapy [220].

Cuproptosis-based and tumor-targeted delivery systems offer promising new strategies for cancer treatment (Table 3). However, numerous scientific complexities remain to be thoroughly investigated prior to clinical application. For instance, although these nanoparticles can inhibit tumor growth, their safety and therapeutic efficacy require further exploration. Accurate tumor targeting is a primary objective when developing new systems. However, given the heterogeneity of tumors, generic delivery strategies may insufficiently address this challenge. Constructing future generations of Cu or its ionophore delivery systems based on specific biomarkers of different tumor types might enhance the system's targeting efficiency. Furthermore, the cost of preparing the delivery system is crucial in determining its scope for clinical application and must be considered in future research.

**Table 3:** Cuproptosis-associated nano-drug delivery systems

Nanoparticle delivery system	Components	Mechanism or strategy of tumor targeting	Function description	References
GOx@(Cu(tz))	Glucose oxidase (GOx), Cu_2_O, 1,2,4-triazole (Htz)	Glucose depletion and GSH stimulation	GOx@(Cu(tz)) can induce cuproptosis in cancer cells under conditions of glucose depletion. Furthermore, GOx@(Cu(tz)) effectively inhibited tumor growth in athymic mice bearing 5637 bladder tumors	[205]
DSF@PEG/Cu-HMSNs	DSF, PEG, Cu^2+^, hollow mesoporous silica nanoparticles (HMSNs)	Mild acidic TME	This nanosystem effectively curbed the growth of 4T1 cell-derived tumors in female BALB/C nude mice	[206]
E-C@DOX NPs	Cu^2+^; ellagic acid (EA); DOX; chondroitin sulfate (CS)	GSH; low-pH environment; CS-mediated internalization	E-C@DOX NPs inhibits tumor cell stemness and cell survival-related pathways, while working in tandem with Cu ions to damage mitochondria and induce cuproptosis, thereby suppressing the ATP-dependent drug efflux pathway and reversing DOX resistance	[207]
LDH/HA/5-FU nanosheets	5-FU; copper–aluminum layered double hydroxide (CuAl-LDH); hyaluronic acid (HA)	pH-responsive; CD44-targeting property of HA	LDH/HA/5-FU nanosheets could rapidly release Cu (II) and 5-FU in tumor cells, and induce tumor cell apoptosis and cuproptosis. LDH/HA/5-FU nanosheets show excellent inhibitory effects on tumors by combining Cu-based chemical dynamics therapy (CDT) and chemotherapy	[208]
PTC	Cu_2_O, AIE photosensitizer (TBP-2), Platelet vesicle (PV),	Acid conditions and hydrogen peroxides; light irradiation	PTC therapy can specifically induce cuproptosis in tumor cells, significantly suppressing the lung metastasis of breast cancer, increasing the population of central memory T cells in peripheral blood, and preventing tumor recurrence	[211]
Au@MSN-Cu/PEG/DSF	Au nanorods (NRs), Cu(NO_3_)_2_, PEG, DSF	PTT	Au@MSN Cu/PEG/DSF can effectively induce tumor cell death and suppress tumor growth in synergy with PTT	[212]
O_2_-PFH@CHPI NPs	Cu^2+^; indocyanine green; O_2_-saturated perfluorohexane (PFH)	pH-Responsive; PTT	Upon NIR, O_2_-PFH@CHPI NPs can simultaneously accelerate catalytic reactions, trigger O^2^ release for PDT to promote oxidative stress, and effectively activate Cu^+^-mediated cuproptosis. Moreover, the tilt of redox balance promotes lipid peroxidation and GPX4 inactivation, resulting in an augmented ferroptosis	[213]
NP@ESCu	Amphiphilic biodegradable polymer (PHPM), ES-Cu	Excessive intracellular ROS	When combined with αPD-L1, NP@ESCu could induce cuproptosis, which enhances the effectiveness of cancer treatment in mouse models with subcutaneous bladder cancer	[214]
CS/MTO-Cu@AMI	Mitoxantrone (MTO), Cu^2+^, amiloride (AMI), chondroitin sulfate (CS),	CS guided CD44 receptor-mediated tumor-specific target; pH/GSH dual-responsive delivery behavior	CS/MTO-Cu@AMI activated the AMPK pathway by inducing cuproptosis and mitochondrial dysfunction, orchestrating the degradation of PD-L1. The treatment-induced dsDNA damage stimulated antitumor immunity through the activation of the cGAS–STING pathway	[215]
CCJD-FA	CaO_2_, Cu^2+^, 3,3′-dithiobis(propionohydrazide) (DTPH), DSPE-PEG-FA, JQ-1	GSH; the acidic condition	CCJD-FA inhibited intracellular glycolysis and ATP production and reduced the expression of IFN-γ-induced PD-L1. This made cancer cells more susceptible to cuproptosis and enhanced immune responses to inhibit tumor growth	[216]
BSO-CAT@MOF-199 @DDM (BCMD)	Butythione sulfoxideimine (BSO), catalase (CAT), dodecyl-β-d-maltoside (DDM), Cu-based MOF of MOF-199	Slightly acidic tumor environment	BCMD-induced cuproptosis of glioblastoma, which in turn triggers immunogenic cell death (ICD) and enhances tumoricidal immunity. Combined with αPD-L1, BCMD substantially enhances the antitumor therapeutic efficiency of immune checkpoint blockade therapy	[217]
Cu-doped BiSex (CBS)	CuI; Bi_2_Se_3_;	PTT	CBS induces cuproptosis and apoptosis and boosts antitumor immune responses during combining with αPD-L1	[218]
CLDCu	Cu^2+^; DSF; low molecular weight heparin-tocopherol succinate (LMWH-TOS); chitosan	Mildly acidic pH condition	CLDCu can trigger enhanced cuproptosis by releasing Cu^2+^ and DSF, and activate STING pathway by releasing chitosan, which potentiates dendritic cells (DCs) maturation and evokes innate and adaptive immunity. CLDCu combined with PD-L1 provokes stronger antitumor immunity	[219]
PEG@Cu_2_O-ES	Cu_2_O; ES; PEG;	PTT/CDT	PEG@Cu_2_O-ES with PTT and CDT effects could generate ROS to attack the ATP-Cu pump, thereby reducing the outflow of Cu ions and aggravating cuproptosis. PEG@Cu_2_O-ES showed strong antitumor effect by inducing cuproptosis, reprogramming TME and thus increasing the response sensitivity to αPD-1	[220]
CuMoO_4_ Nanodots	Cu^2+^, MoO_4_^2−^, SDS	PTT	Under sustained PTT, CuMoO_4_ can effectively induce both cuproptosis and ferroptosis in tumor cells, thereby triggering an immune response to ICD	[221]
CSTD-Cu(II)@DSF	Generation 5 (G5), phenylboronic acid (PBA), mannose, Cu^2+^, DSF	Low pH; high ROS	CSTD-Cu (II)@DSF showed significant potential in suppressing the growth of MCF7 tumors by integrating chemotherapy with cuproptosis and CDT. Moreover, it allows for T1-weighted real-time MR imaging of tumors in vivo	[222]
Au NCs-Cu^2+^@SA-HA NHGs	NAC, 4-mercaptobenzoic acid (4-MBA), HAuCl_4_, NaOH, NaBH_4_, CuCl_2_,	Excessive existence of H^+^ ions; PTT; PDT	The use of Au25(NAMB)18 NCs-Cu^2+^@SA/HA NHGs can significantly enhance the efficacy of cuproptosis-based tumor therapy by depleting the overexpressed GSH and H_2_O_2_ in the TME. Simultaneously, Au25(NAMB)18 NCs-Cu^2+^@SA/HA can be employed for imaging-guided diagnosis and treatment of tumors	[223]
Cu2O@CuBTC-DSF@HA nanocomposites (CCDHs)	Cu_2_O, trimesic acid (H_3_BTC), DSF, hyaluronic acid (HA)	Acidic environment	CCDHs synergistically enhanced cuproptosis rather than trigger apoptosis, exhibiting superior anti-tumor effectiveness and minimal toxicity	[224]
SonoCu	Cu^2+^, zeolitic imidazolate framework-8, perfluorocarbon (PFC), chlorin e6 (Ce6), O_2_	Sonodynamic therapy	SonoCu activated sonodynamic cuproptosis, showing cytotoxicity against cancer cells but sparing normal cells in response to ultrasound treatment	[225]
CuX-P	PD-1-overexpressing T cell membrane, Mxene, Cu^2+^, DSF	PD-1	CuX-P can bind with and deplete PD-L1 on the surface of tumor cells, promoting its endocytosis. This action may trigger cuproptosis in tumor cells, thereby intensifying the antitumor immune responses in TNBC	[226]
DMMA@Cu_2_-xSe	Poly(ethylene imine) (PEI), 2,3-dimethylmaleic anhydride (DMMA), Cu_2_-xSe, RGD polypeptide	The weak, acidic environment	DMMA@Cu_2_-xSe displayed the ability to enhance thermotherapy through cuproptosis-driven mechanisms	[227]
ART@CuT/ETH HNP	3,3′-Dithiobis(propionohydrazide) (TPH), Cu^2+^, DSF, hyaluronan (HAT), artemisinin (ART)	Acidic and GSH-rich intracellular microenvironment	ART@CuT/ETH HNP effectively triggered cancer cell death through a synergistic combination of cuproptosis, apoptosis, and ferroptosis	[228]
ZIF-8-Cu_2_O-DNA	ZIF-8, Zn^2+^, Cu_2_O, DNA	The weak, acidic environment	ZIF-8-Cu_2_O-DNA was able to increase ROS and enhance cuproptosis, thereby inhibiting tumor growth by synergizing cuproptosis with genic and CDT	[229]
CuET NPs	Bovine serum albumin (BSA), CuET, sodium diethyldithiocarbamate trihydrate (NaDTC)	Not described	CuET NPs notably induced cuproptosis in A549/DDP cells and effectively inhibited tumor growth in a cisplatin-resistant tumor model, demonstrating superior biosafety	[230]
TP-M–Cu–MOF/siATP7a	Copper-based metal–organic frameworks (Cu-MOF), siRNA targeting ATP7a (siATP7a), TP0751 peptide appended stem cell membrane (TPM)	TP0751 peptide decorated mesenchymal stem cell membrane	TP-M–Cu–MOF/siATP7a efficiently silenced the ATP7A gene and increased copper intake, thereby inducing cuproptosis and enhancing therapeutic efficacy in small-cell lung cancer brain metastasis tumor-bearing mice	[231]
Cu-GA NPs	Cu^2+^, gallic acid (GA), polyvinylpyrrolidone (PVP)	Highly expressed GSH in tumor cells	Cu-GA NPs significantly depleted intracellular GSH and increased ROS levels, resulting in severe cell cuproptosis and apoptosis. In vivo experiments demonstrated that Cu-GA NPs effectively suppressed tumor growth through a combination of chemotherapy and CDT	[232]
HFn-Cu-REGO NPs	Human heavy-chain ferritin (HFn), Cu^2+^, Regorafenib	HFn guided GBM accumulation; pH-responsive delivery behavior	HFn-Cu-REGO NPs exhibited excellent GBM suppression in vivo due to their ability to block autophagic flux and induce cuproptosis	[233]
Cu_2_(PO_4_)(OH) NPs	Cu_2_(PO_4_)(OH)	H_2_S-induced copper overload in tumor TME	Cu_2_(PO_4_)(OH) NPs inhibited tumor cell growth by triggering cuproptosis and pyroptosis	[234]
Cu-LDH	Layered double hydroxide (LDH) nanoparticle; Cu^2+^	Tumor site injection; acidic condition responsive	Cu-LDH nanoparticles, as lysosome destroyer, enhance Cu-mediated cuproptosis and pyroptosis for high-efficiency cancer immunotherapy	[235]
Cu-DBCO/CL	Cu-dibenzo-[g,p]chrysene-2,3,6,7,10,11,14,15-octaol (DBCO); cholesterol oxidase (CHO); lysyl oxidase inhibitor (LOX-IN-3); 2,2′-[propane-2,2-diylbis(thio)]diacetic acid linker (PSDA)	ROS-responsive	Cu-DBCO/CL triggers tumor cell cuproptosis and ferroptosis, simultaneously enhances ICD of cancer cells and reinvents the ECM, leading to a potent inhibition of tumor growth and metastasis	[236]
OMP	2-(*N*-oxide-*N*,*N*-diethylamino)ethyl methacrylate (OPDEA); 2-methylimidazole; Cu(NO_+_)_2_; Zn(NO3)_2_⋅6H_2_O; siPDK	OPDEA-mediated pulmonary mucosa penetration	siPDK released from OMP sensitizes the cuproptosis by inhibiting intracellular glycolysis, ATP production, and blocking the Cu^+^ efflux protein ATP7B. OMP-mediated cuproptosis triggers ICD to promote DC maturation and CD8^+^ T cells infiltration, upregulates membrane-associated PD-L1 expression and induces soluble PD-L1 secretion. OMP combined with aPD-L1 afford preferable efficacy against lung metastasis	[237]
MCD	Dendritic mesoporous silica nanoparticles (MSN); Cu_2_S; oxidized dextran (oDEX)	pH-responsive; PTT	MCD triggers tumor cell cuproptosis by inhibiting key proteins in TCA cycle. MCD effectively mitigates tumor growth and osteosarcoma-induced bone destruction in vivo under NIR-II light irradiation	[238]
CuET@PH NPs	Polydopamine; hydroxyethyl starch; copper-diethyldithiocarbamate (CuET)	Hyperbaric oxygen (HBO)	The combination of HBO and CuET@PH NPs potently suppresses energy metabolism of cancer stem cells, thereby achieving robust tumor inhibition of PDAC and significantly elongating tumor mice survival	[239]
PCD@CM	NIR-II ultrasmall polymer dots; Cu^2+^; DOX; 4T1 cell membrane	GSH-responsive; homotypic cancer cell membrane-mediated self-recognition and internalization; PTT	Cu released by PCD@CM induces the aggregation of lipoylated mitochondrial proteins accompanied by the loss of iron–sulfur proteins, leading to severe proteotoxic stress and eventually cuproptosis. NIR-II PTT and GSH depletion render tumor cells more sensitive to cuproptosis. The amplified cuproptosis significantly sensitized aPD-L1-mediated tumor immunotherapy	[240]
D-CuxOS@Fe-MOF	Cu^2+^; Fe^3+^; d-/l-penicillamine; NH2-BDC	pH-responsive	D-CuxOS@Fe-MOF induces augmented oxidative stress and potent ferroptosis, which synergizes with cuproptosis for enhanced cancer therapy	[241]
T-HCN@CuMS	Heterogeneous carbon nitride (HCN); Cu-loaded metallic molybdenum bisulfide nanosheets (CuMS); cRGDfk-PEG2k-DSPE	cRGDfk-PEG2k-DSPE-mediated specific recognition to αvβ3 integrins of tumor cells; PTT	T-HCN@CuMS presents a favorable photo-induced catalytic property to generate abundant ROS under NIR. It efficiently catalyzes the Fenton-like reaction and triggers cell cuproptosis, resulting in favorable therapeutic outcomes to inhibit tumor growth and metastasis	[242]
CQG NPs	Cu^2+^; polyvinylpyrrolidone (PVP); gallic acid; (3-aminopropyl) triethoxysilane (APTES); GOx	GSH-responsive	CQG NPs can induce cuproptosis by released Cu and depletion of GSH, and induce pyroptosis by disrupting the antioxidant defense mechanism of tumor cells, thereby enhancing the infiltration of immune cells into the tumor and activating robust systemic immunity	[243]

### Cuproptosis and platinum resistance

Treatment with platinum drugs is a standard of care for different tumor types. Studies have reported that Cu metabolism and cuproptosis have an important impact on the treatment of tumors with platinum-based drugs. First, some genes related to Cu metabolism or cuproptosis can influence tumor cell sensitivity to platinum drugs. For instance, GLS activity upregulated in cisplatin-resistant CRC cell lines, which reduced the cells’ sensitivity to cisplatin [244]. Further investigation revealed that the binding of YTHDF1 to the 3′ UTR of GLS1 promoted GLS protein synthesis, which mediated the cisplatin resistance of CRC cells [244].

Moreover, it has been reported that Cu transporters were involved in platinum uptake or expulsion. SLC31A1 has been recognized as the primary transporter of platinum-based drugs such as cisplatin [245]. Research by Wu et al. revealed that in epithelial ovarian cancer cells, ZNF711, a type of zinc-finger protein, could recruit histone demethylase JHDM2A to the *SLC31A1* promoter [246]. This action served to decrease the H3K9me2 level, thus activating *SLC31A1* transcription and enhancing cisplatin uptake. Moreover, Cheng et al. reported that, in cisplatin-resistant osteosarcoma, the RNA-binding protein PTBP1 accelerated the *SLC31A1* mRNA degradation by directly binding to it, which downregulated the SLC31A1 protein level and reduced the cell’s sensitivity to cisplatin [247].

Besides, ATP7A and ATP7B can interact with platinum drugs and pump them across membranes, contributing to the resistance of tumor cells to platinum-based cancer treatments. Targeting ATP7A/ATP7B could increase tumor cell sensitivity to platinum drugs [248–253]. Additionally, Ryumon et al. found that the Cu chelator ammonium tetrathiomolybdate could downregulate ATP7B expression in HNSCC cells in vitro, thereby enhancing the anticancer effect of cisplatin in a bone invasion mouse model [254]. This implies that altering the Cu levels of tumor cells could be a viable approach to modulate ATP7B expression and counter cancer cell resistance to cisplatin. These studies indicate that, while the expression levels of Cu transporters may be unfavorable for the prognosis of patients with cancer, they could serve as potential biomarkers for therapy associated with platinum drugs. Future research should focus on elucidating the mechanisms that underpin the relationship between Cu absorption and resistance to platinum-based drugs.

Moreover, the potential of Cu ionophore-induced cuproptosis to overcome platinum drug resistance in tumor cells is noteworthy. For instance, ES has been demonstrated to enhance the sensitivity of lung cancer cells to cisplatin [33]. The upregulation of TRX-1, a small redox protein that exhibits an inverse relationship with the intensity of mitochondrial respiration in cisplatin-resistant cells, might serve as the primary mechanism [33]. However, in clinical practice, whether ES can benefit patients with cisplatin-resistant lung cancer remains an uncertain issue. In cervical cancer cells, the DSF/Cu complex can decrease the population of cancer stem cell-like LGR5^+^ cells, which mediates cisplatin resistance in tumor cells [255]. Furthermore, it has been reported that combined treatment with DSF and cisplatin can alter the cellular localization of ATP7A, thereby increasing the drug concentration within tumor cells, promoting apoptosis, and inhibiting tumor growth [256]. Targeted delivery of Cu to tumor cells using nanoparticle systems, like the CuET NPs system designed by Lu et al., could potentially counteract the phenomenon of drug resistance in tumor treatment [230]. This system has been demonstrated to effectively inhibit the growth of cisplatin-resistant lung cancer cells in vivo and in vitro. However, these studies are predicated on the premise of Cu overload in tumor cells, and the specific role that cuproptosis plays in tumor drug resistance is not yet clearly defined. Future research focusing on the influence of the cuproptosis pathway on tumor drug resistance could provide valuable insights that may help address current challenges in clinical treatment.

## Conclusions and future perspectives

Cu-mediated cell death was discovered about four decades ago, and the induction of cell death by Cu and Cu ionophores, such as ES and DSF, has been extensively researched in tumor therapy. However, the molecular mechanism underlying cell death triggered by intracellular Cu overload remained unclear until the concept of “cuproptosis” emerged. Tsvetkov et al. identified that the increase in Cu levels led to cell death and established that this was reliant on the Cu-induced DLAT aggregation and Fe–S cluster protein instability. Using systematic screening, they also identified several crucial regulatory genes involved in cuproptosis [7]. While TCA-related proteins like FDX1 and DLAT are presently employed as biomarkers in cuproptosis research, investigations into the interplay between TCA and its elements with cuproptosis are still limited, particularly regarding the molecular mechanisms underlying cuproptosis induced by TCA elements. As a novel form of cell death, cuproptosis still lacks reliable biomarkers for identification and evaluation, limiting physiological and pathological studies. This is a barrier to understanding the full potential and implications of cuproptosis in biological contexts.

Cuproptosis, as an emergent form of cell death, is being actively explored in various areas, including tumor chemotherapy, microenvironment infiltration, immunotherapy, and prognostic evaluation, with the aim of devising more potent cancer treatment strategies. Nonetheless, numerous challenges must be addressed before cuproptosis can be effectively integrated into clinical cancer treatments. For example, the current inability to precisely differentiate the mechanisms and induction approaches of cuproptosis in normal versus cancer cells could potentially compromise treatment precision and exacerbate side effects during cancer therapy. Research on cuproptosis-based cancer therapy can take two main directions. First, the unique traits of cancer cells, including enhanced mitochondrial metabolism and elevated ROS levels, can be leveraged to augment the anticancer efficiency of cuproptosis-associated drugs or systems. Second, the possibility of inhibiting cuproptosis regulatory proteins or pathways, such as MTF1, GLS, and CDKN2A, can be examined to enhance or sensitize cuproptosis in cancer cells. Additionally, the heterogeneous nature of tumors results in varying responses of tumor cells to Cu ions and, consequently, to cuproptosis. Therefore, conducting a comprehensive study of the molecular mechanisms or regulatory networks involved in Cu metabolism, and the resulting cell damage across different cancer cells could potentially broaden the applicability of cuproptosis in cancer therapy. Additionally, the prolonged intake of nontargeted Cu or Cu ionophores can disrupt a patient’s mental balance and heighten treatment side effects. An effective approach to mitigate this issue could be the use of a nanoparticle-based targeted delivery system, which can decrease the accumulation of nonessential Cu ions in the body and enhance tumor treatment efficacy. This presents a promising direction for the future development of cuproptosis-related cancer therapeutic strategies. Nonetheless, it is crucial to consider reports suggesting that elevated Cu concentrations can promote the progression of certain tumors. Hence, achieving a balance between the dual effects of Cu is fundamental for implementing cuproptosis-based cancer treatment.

In summary, cuproptosis represents a significant new form of cell death, providing potential direction and strategies for cancer therapy. However, we are still in the early stages of understanding this process, and further research is required to fully elucidate its molecular mechanisms and their links to cancer.

## Data Availability

Not applicable.

## Abbreviations

LA: Lipoic acid
TCA: Tricarboxylic acid
ES: Elesclomol
DSF: Disulfram
CTR1: Copper transporter 1
SLC31A1: Solute carrier family 31 member 1
STEAP: Six-transmembrane epithelial antigen of the prostate
ATP7A: ATPase copper transporter α
ATP7B: ATPase copper transporter β
TGN: Trans-Golgi network
MT1/2: Metallothionein 1/2
GSH: Glutathione
ATOX1: Antioxidant 1
COX: Cytochrome oxidase
SOD1: Superoxide dismutase 1
IMM: Inner mitochondrial membrane
IMS: Intermembrane space
SCO1: Synthesis of cytochrome c oxidase 1
SLC25A3: Solute carrier family 25 member 3
ROS: Reactive oxygen species
HCC: Hepatocellular carcinoma
NAC: *N*-acetylcysteine
FDX1: Ferredoxin 1
LIAS: LA synthase
LIPT1: Lipoyl transferase 1
DLAT: Drolipoamide *S*-acetyltransferase
DLD: Dihydrolipoamide dehydrogenase
PDHA1: Pyruvate dehydrogenase E1 subunit alpha 1
PDHB: Pyruvate dehydrogenase E1 subunit beta
MTF1: Metal-regulatory transcription factor-1
GLS: Glutaminase
CDKN2A: Cyclin-dependent kinase inhibitor 2A
G6PD: Glucose-6-phosphate dehydrogenase
RCC: Renal cell carcinoma
FOXO3: Forkhead box O3
GPX4: Glutathione peroxidase 4
JNK: Jun N-terminal kinase
MEK1: Mitogen-activated extracellular signal-regulated kinase 1
MAPK: Mitogen-activated protein kinase
ERK1/2: Extracellular signal-regulated kinase 1/2
OCSCC: Oral cavity squamous cell carcinoma
NADPH: Nicotinamide adenine dinucleotide phosphate
CK2: Casein kinase II
ULK: Unc-51-like autophagy-activating kinase
AMPK: Adenosine 5′-monophosphate (AMP)-activated protein kinase
mTOR: Mammalian target of rapamycin
ATG4B: Autophagy-related gene 4B
VEGF: Vascular endothelial growth factor
FGF: Fibroblast growth factor
LOX: Lysyl oxidase
TNBC: Triple-negative breast cancer
PD-L1: Programmed death-ligand 1
LUAD: Lung adenocarcinoma
KIRC: Kidney renal clear-cell carcinoma
PD-1: Programmed death-1
PDC: Pyruvate dehydrogenase complex
READ: Rectum adenocarcinoma
NSCLC: Non-small-cell lung cancer
PAAD: Pancreatic adenocarcinoma
SNAP25: Synaptosomal-associated protein 25
PCA: Prostate cancer
CDK: Cyclin-dependent kinases
HNSCC: Head and neck squamous cell carcinoma
MT: Metallothionein
LGG: Lower-grade glioma
CRG: Cuproptosis-related gene
CRGI: Cuproptosis-related gene index
LDH: Lactate dehydrogenase
CRIRS: Cuproptosis-related immune risk score
DOX: Doxorubicin
PTT: Photothermal therapy
NIR: Near-infrared radiation
αPD-L1: Anti-PD-L1 antibody
PFS: Progression-free survival
OS: Overall survival
CRC: Colorectal cancer
ACC: Adrenocortical carcinoma
READ: Rectum adenocarcinoma
LIHC: Liver hepatocellular carcinoma
ESCA: Esophageal carcinoma
BRCA: Breast invasive carcinoma
COAD: Colon adenocarcinoma
KIPAN: Pan-kidney cohort
KIRP: Kidney renal papillary cell carcinoma
STAD: Stomach adenocarcinoma
OSCC: Oral squamous cell carcinoma
PI: Proteasome inhibitors
CuET: Bis-diethyldithiocarbamate-copper
GOx: Glucose oxidase
TME: Tumor microenvironment
ECM: Extracellular matrix
ICD: Immunogenic cell death (ICD)
CDT: Chemical dynamics
PDT: Photodynamic therapy
DCs: Dendritic cells

## References

[1] Festa RA, Thiele DJ (2011). Copper: an essential metal in biology. Curr Biol.

[2] Chen L, Min J, Wang F (2022). Copper homeostasis and cuproptosis in health and disease. Signal Transduct Target Ther.

[3] Halliwell B, Gutteridge JM (1984). Oxygen toxicity, oxygen radicals, transition metals and disease. Biochem J.

[4] Kirshner JR, He S, Balasubramanyam V, Kepros J, Yang CY (2008). Elesclomol induces cancer cell apoptosis through oxidative stress. Mol Cancer Ther.

[5] Oliveri V (2022). Selective targeting of cancer cells by copper ionophores: an overview. Front Mol Biosci.

[6] Zheng P, Zhou C, Lu L, Liu B, Ding Y (2022). Elesclomol: a copper ionophore targeting mitochondrial metabolism for cancer therapy. J Exp Clin Cancer Res.

[7] Tsvetkov P, Coy S, Petrova B, Dreishpoon M, Verma A, Abdusamad M (2022). Copper induces cell death by targeting lipoylated TCA cycle proteins. Science.

[8] Ge EJ, Bush AI, Casini A, Cobine PA, Cross JR, DeNicola GM (2022). Connecting copper and cancer: from transition metal signalling to metalloplasia. Nat Rev Cancer.

[9] Finney L, Vogt S, Fukai T, Glesne D (2009). Copper and angiogenesis: unravelling a relationship key to cancer progression. Clin Exp Pharmacol Physiol.

[10] Gupte A, Mumper RJ (2009). Elevated copper and oxidative stress in cancer cells as a target for cancer treatment. Cancer Treat Rev.

[11] Cobine PA, Brady DC (2022). Cuproptosis: cellular and molecular mechanisms underlying copper-induced cell death. Mol Cell.

[12] Xie J, Yang Y, Gao Y, He J (2023). Cuproptosis: mechanisms and links with cancers. Mol Cancer.

[13] Wang Y, Chen Y, Zhang J, Yang Y, Fleishman JS, Wang Y (2024). Cuproptosis: a novel therapeutic target for overcoming cancer drug resistance. Drug Resist Updat.

[14] Lönnerdal B (2008). Intestinal regulation of copper homeostasis: a developmental perspective. Am J Clin Nutr.

[15] Wee NK, Weinstein DC, Fraser ST, Assinder SJ (2013). The mammalian copper transporters CTR1 and CTR2 and their roles in development and disease. Int J Biochem Cell Biol.

[16] Knutson MD (2007). Steap proteins: implications for iron and copper metabolism. Nutr Rev.

[17] Wyman S, Simpson RJ, McKie AT, Sharp PA (2008). Dcytb (Cybrd1) functions as both a ferric and a cupric reductase in vitro. FEBS Lett.

[18] La Fontaine S, Ackland ML, Mercer JF (2010). Mammalian copper-transporting P-type ATPases, ATP7A and ATP7B: emerging roles. Int J Biochem Cell Biol.

[19] Lutsenko S, Barnes NL, Bartee MY, Dmitriev OY (2007). Function and regulation of human copper-transporting ATPases. Physiol Rev.

[20] Tsang T, Davis CI, Brady DC (2021). Copper biology. Curr Biol.

[21] Polishchuk R, Lutsenko S (2013). Golgi in copper homeostasis: a view from the membrane trafficking field. Histochem Cell Biol.

[22] Liu N, Lo LS, Askary SH, Jones L, Kidane TZ, Trang T (2007). Transcuprein is a macroglobulin regulated by copper and iron availability. J Nutr Biochem.

[23] Moriya M, Ho YH, Grana A, Nguyen L, Alvarez A, Jamil R (2008). Copper is taken up efficiently from albumin and alpha2-macroglobulin by cultured human cells by more than one mechanism. Am J Physiol Cell Physiol.

[24] Freedman JH, Ciriolo MR, Peisach J (1989). The role of glutathione in copper metabolism and toxicity. J Biol Chem.

[25] Hamza I, Prohaska J, Gitlin JD (2003). Essential role for Atox1 in the copper-mediated intracellular trafficking of the Menkes ATPase. Proc Natl Acad Sci USA.

[26] Garza NM, Swaminathan AB, Maremanda KP, Zulkifli M, Gohil VM (2023). Mitochondrial copper in human genetic disorders. Trends Endocrinol Metab.

[27] Boulet A, Vest KE, Maynard MK, Gammon MG, Russell AC, Mathews AT (2018). The mammalian phosphate carrier SLC25A3 is a mitochondrial copper transporter required for cytochrome c oxidase biogenesis. J Biol Chem.

[28] Cen D, Brayton D, Shahandeh B, Meyskens FL, Farmer PJ (2004). Disulfiram facilitates intracellular Cu uptake and induces apoptosis in human melanoma cells. J Med Chem.

[29] Tardito S, Bassanetti I, Bignardi C, Elviri L, Tegoni M, Mucchino C (2011). Copper binding agents acting as copper ionophores lead to caspase inhibition and paraptotic cell death in human cancer cells. J Am Chem Soc.

[30] Allensworth JL, Evans MK, Bertucci F, Aldrich AJ, Festa RA, Finetti P (2015). Disulfiram (DSF) acts as a copper ionophore to induce copper-dependent oxidative stress and mediate anti-tumor efficacy in inflammatory breast cancer. Mol Oncol.

[31] Nagai M, Vo NH, Shin Ogawa L, Chimmanamada D, Inoue T, Chu J (2012). The oncology drug elesclomol selectively transports copper to the mitochondria to induce oxidative stress in cancer cells. Free Radic Biol Med.

[32] Buccarelli M, D'Alessandris QG, Matarrese P, Mollinari C, Signore M, Cappannini A (2021). Elesclomol-induced increase of mitochondrial reactive oxygen species impairs glioblastoma stem-like cell survival and tumor growth. J Exp Clin Cancer Res.

[33] Wangpaichitr M, Wu C, You M, Maher JC, Dinh V, Feun LG (2009). Nʹ, Nʹ-dimethyl-Nʹ, Nʹ-bis(phenylcarbonothioyl) propanedihydrazide (elesclomol) selectively kills cisplatin resistant lung cancer cells through reactive oxygen Species (ROS). Cancers.

[34] Yang Z, Guo F, Albers AE, Sehouli J, Kaufmann AM (2019). Disulfiram modulates ROS accumulation and overcomes synergistically cisplatin resistance in breast cancer cell lines. Biomed Pharmacother.

[35] Swetha KL, Sharma S, Chowdhury R, Roy A (2020). Disulfiram potentiates docetaxel cytotoxicity in breast cancer cells through enhanced ROS and autophagy. Pharmacol Rep.

[36] Morrison BW, Doudican NA, Patel KR, Orlow SJ (2010). Disulfiram induces copper-dependent stimulation of reactive oxygen species and activation of the extrinsic apoptotic pathway in melanoma. Melanoma Res.

[37] Liu Y, Guan X, Wang M, Wang N, Chen Y, Li B (2022). Disulfiram/Copper induces antitumor activity against gastric cancer via the ROS/MAPK and NPL4 pathways. Bioengineered.

[38] Shah O'Brien P, Xi Y, Miller JR, Brownell AL, Zeng Q, Yoo GH (2019). Disulfiram (Antabuse) oxctivates ROS-dependent ER stress and apoptosis in oral cavity squamous cell carcinoma. J Clin Med.

[39] Chiba T, Suzuki E, Yuki K, Zen Y, Oshima M, Miyagi S (2014). Disulfiram eradicates tumor-initiating hepatocellular carcinoma cells in ROS-p38 MAPK pathway-dependent and -independent manners. PLoS ONE.

[40] Modica-Napolitano JS, Bharath LP, Hanlon AJ, Hurley LD (2019). The anticancer agent elesclomol has direct effects on mitochondrial bioenergetic function in Isolated mammalian mitochondria. Biomolecules.

[41] Chen SY, Chang YL, Liu ST, Chen GS, Lee SP, Huang SM (2021). Differential cytotoxicity mechanisms of copper complexed with disulfiram in oral cancer cells. Int J Mol Sci.

[42] Lee JH, Cho YS, Jung KH, Park JW, Lee KH (2020). Genipin enhances the antitumor effect of elesclomol in A549 lung cancer cells by blocking uncoupling protein-2 and stimulating reactive oxygen species production. Oncol Lett.

[43] Ren Y, Lin Y, Chen J, Jin Y (2021). Disulfiram chelated with copper promotes apoptosis in osteosarcoma via ROS/mitochondria pathway. Biol Pharm Bull.

[44] Xu Y, Zhou Q, Feng X, Dai Y, Jiang Y, Jiang W (2020). Disulfiram/copper markedly induced myeloma cell apoptosis through activation of JNK and intrinsic and extrinsic apoptosis pathways. Biomed Pharmacother.

[45] Hassani S, Ghaffari P, Chahardouli B, Alimoghaddam K, Ghavamzadeh A, Alizadeh S (2018). Disulfiram/copper causes ROS levels alteration, cell cycle inhibition, and apoptosis in acute myeloid leukaemia cell lines with modulation in the expression of related genes. Biomed Pharmacother.

[46] Guo W, Zhang X, Lin L, Wang H, He E, Wang G (2021). The disulfiram/copper complex induces apoptosis and inhibits tumour growth in human osteosarcoma by activating the ROS/JNK signalling pathway. J Biochem.

[47] Gao W, Huang Z, Duan J, Nice EC, Lin J, Huang C (2021). Elesclomol induces copper-dependent ferroptosis in colorectal cancer cells via degradation of ATP7A. Mol Oncol.

[48] Ren X, Li Y, Zhou Y, Hu W, Yang C, Jing Q (2021). Overcoming the compensatory elevation of NRF2 renders hepatocellular carcinoma cells more vulnerable to disulfiram/copper-induced ferroptosis. Redox Biol.

[49] Li Y, Chen F, Chen J, Chan S, He Y, Liu W (2020). Disulfiram/copper induces antitumor activity against both nasopharyngeal cancer cells and cancer-associated fibroblasts through ROS/MAPK and ferroptosis pathways. Cancers.

[50] Chu M, An X, Fu C, Yu H, Zhang D, Li Q (2023). Disulfiram/copper induce ferroptosis in triple-negative breast cancer cell Line MDA-MB-231. Front Biosci.

[51] Li C, Zhou S, Chen C, Zhu L, Li S, Song Z (2023). DDTC-Cu(I) based metal-organic framework (MOF) for targeted melanoma therapy by inducing SLC7A11/GPX4-mediated ferroptosis. Coll Surf B Biointerfaces.

[52] Hu Y, Qian Y, Wei J, Jin T, Kong X, Cao H (2021). The Disulfiram/copper complex induces autophagic cell death in colorectal cancer by targeting ULK1. Front Pharmacol.

[53] Park YM, Go YY, Shin SH, Cho JG, Woo JS, Song JJ (2018). Anti-cancer effects of disulfiram in head and neck squamous cell carcinoma via autophagic cell death. PLoS ONE.

[54] Guo F, Yang Z, Kulbe H, Albers AE, Sehouli J, Kaufmann AM (2019). Inhibitory effect on ovarian cancer ALDH+ stem-like cells by Disulfiram and Copper treatment through ALDH and ROS modulation. Biomed Pharmacother.

[55] Vallières C, Holland SL, Avery SV (2017). Mitochondrial ferredoxin determines vulnerability of cells to copper excess. Cell Chem Biol.

[56] Rowland EA, Snowden CK, Cristea IM (2018). Protein lipoylation: an evolutionarily conserved metabolic regulator of health and disease. Curr Opin Chem Biol.

[57] Sheftel AD, Stehling O, Pierik AJ, Elsässer HP, Mühlenhoff U, Webert H (2010). Humans possess two mitochondrial ferredoxins, Fdx1 and Fdx2, with distinct roles in steroidogenesis, heme, and Fe/S cluster biosynthesis. Proc Natl Acad Sci USA.

[58] Tort F, Ferrer-Cortès X, Thió M, Navarro-Sastre A, Matalonga L, Quintana E (2014). Mutations in the lipoyltransferase LIPT1 gene cause a fatal disease associated with a specific lipoylation defect of the 2-ketoacid dehydrogenase complexes. Hum Mol Genet.

[59] Joshi PR, Sadre S, Guo XA, McCoy JG, Mootha VK (2023). Lipoylation is dependent on the ferredoxin FDX1 and dispensable under hypoxia in human cells. J Biol Chem.

[60] Kastaniotis AJ, Autio KJ, Kerätär JM, Monteuuis G, Mäkelä AM, Nair RR (2017). Mitochondrial fatty acid synthesis, fatty acids and mitochondrial physiology. Biochim Biophys Acta Mol Cell Biol Lipids.

[61] Patel MS, Nemeria NS, Furey W, Jordan F (2014). The pyruvate dehydrogenase complexes: structure-based function and regulation. J Biol Chem.

[62] Patel MS, Korotchkina LG (2006). Regulation of the pyruvate dehydrogenase complex. Biochem Soc Trans.

[63] Günther V, Lindert U, Schaffner W (2012). The taste of heavy metals: gene regulation by MTF-1. Biochim Biophys Acta.

[64] Márquez J, de la Oliva AR, Matés JM, Segura JA, Alonso FJ (2006). Glutaminase: a multifaceted protein not only involved in generating glutamate. Neurochem Int.

[65] Ruas M, Peters G (1998). The p16INK4a/CDKN2A tumor suppressor and its relatives. Biochim Biophys Acta.

[66] Tadini-Buoninsegni F, Smeazzetto S (2017). Mechanisms of charge transfer in human copper ATPases ATP7A and ATP7B. IUBMB Life.

[67] Tümer Z, Møller LB (2010). Menkes disease. Eur J Hum Genet.

[68] Członkowska A, Litwin T, Dusek P, Ferenci P, Lutsenko S, Medici V (2018). Wilson disease. Nat Rev Dis Primers.

[69] Kim H, Wu X, Lee J (2013). SLC31 (CTR) family of copper transporters in health and disease. Mol Aspects Med.

[70] Lu J, Ling X, Sun Y, Liu L, Liu L, Wang X (2023). FDX1 enhances endometriosis cell cuproptosis via G6PD-mediated redox homeostasis. Apoptosis.

[71] Sun L, Zhang Y, Yang B, Sun S, Zhang P, Luo Z (2023). Lactylation of METTL16 promotes cuproptosis via m^6^A-modification on FDX1 mRNA in gastric cancer. Nat Commun.

[72] Wang X, Jia JH, Zhang M, Meng QS, Yan BW, Ma ZY (2023). Adrenomedullin/FOXO3 enhances sunitinib resistance in clear cell renal cell carcinoma by inhibiting FDX1 expression and cuproptosis. FASEB J.

[73] Wang W, Lu K, Jiang X, Wei Q, Zhu L, Wang X (2023). Ferroptosis inducers enhanced cuproptosis induced by copper ionophores in primary liver cancer. J Exp Clin Cancer Res.

[74] Xue Q, Yan D, Chen X, Li X, Kang R, Klionsky DJ (2023). Copper-dependent autophagic degradation of GPX4 drives ferroptosis. Autophagy.

[75] Jiang X, Stockwell BR, Conrad M (2021). Ferroptosis: mechanisms, biology and role in disease. Nat Rev Mol Cell Biol.

[76] Xue Q, Kang R, Klionsky DJ, Tang D, Liu J, Chen X (2023). Copper metabolism in cell death and autophagy. Autophagy.

[77] Redza-Dutordoir M, Averill-Bates DA (2016). Activation of apoptosis signaling pathways by reactive oxygen species. Biochim Biophys Acta.

[78] Wang T, Chen X, Long X, Liu Z, Yan S (2016). Copper nanoparticles and copper sulphate induced cytotoxicity in hepatocyte primary cultures of *Epinephelus coioides*. PLoS ONE.

[79] Wang T, Long X, Liu Z, Cheng Y, Yan S (2015). Effect of copper nanoparticles and copper sulphate on oxidation stress, cell apoptosis and immune responses in the intestines of juvenile *Epinephelus coioides*. Fish Shellfish Immunol.

[80] Wu H, Guo H, Liu H, Cui H, Fang J, Zuo Z (2020). Copper sulfate-induced endoplasmic reticulum stress promotes hepatic apoptosis by activating CHOP, JNK and caspase-12 signaling pathways. Ecotoxicol Environ Saf.

[81] Xie F, Peng F (2021). Reduction in copper uptake and inhibition of prostate cancer cell proliferation by novel steroid-based compounds. Anticancer Res.

[82] Liu H, Zhang Y, Zheng S, Weng Z, Ma J, Li Y (2016). Detention of copper by sulfur nanoparticles inhibits the proliferation of A375 malignant melanoma and MCF-7 breast cancer cells. Biochem Biophys Res Commun.

[83] Turski ML, Thiele DJ (2009). New roles for copper metabolism in cell proliferation, signaling, and disease. J Biol Chem.

[84] Nasulewicz A, Mazur A, Opolski A (2004). Role of copper in tumour angiogenesis–clinical implications. J Trace Elem Med Biol.

[85] Ramchandani D, Berisa M, Tavarez DA, Li Z, Miele M, Bai Y (2021). Copper depletion modulates mitochondrial oxidative phosphorylation to impair triple negative breast cancer metastasis. Nat Commun.

[86] MacDonald G, Nalvarte I, Smirnova T, Vecchi M, Aceto N, Dolemeyer A (2014). Memo is a copper-dependent redox protein with an essential role in migration and metastasis. Sci Signal.

[87] Shanbhag V, Jasmer-McDonald K, Zhu S, Martin AL, Gudekar N, Khan A (2019). ATP7A delivers copper to the lysyl oxidase family of enzymes and promotes tumorigenesis and metastasis. Proc Natl Acad Sci USA.

[88] Yücel I, Arpaci F, Ozet A, Döner B, Karayilanoğlu T, Sayar A (1994). Serum copper and zinc levels and copper/zinc ratio in patients with breast cancer. Biol Trace Elem Res.

[89] Feng Y, Zeng JW, Ma Q, Zhang S, Tang J, Feng JF (2020). Serum copper and zinc levels in breast cancer: a meta-analysis. J Trace Elem Med Biol.

[90] Duan F, Li J, Huang J, Hua X, Song C, Wang L (2021). Establishment and validation of prognostic nomograms based on serum copper level for patients with early-stage triple-negative breast cancer. Front Cell Dev Biol.

[91] Zhang L, Shao J, Tan SW, Ye HP, Shan XY (2022). Association between serum copper/zinc ratio and lung cancer: a systematic review with meta-analysis. J Trace Elem Med Biol.

[92] Zhang X, Yang Q (2018). Association between serum copper levels and lung cancer risk: a meta-analysis. J Int Med Res.

[93] Zhang M, Shi M, Zhao Y (2018). Association between serum copper levels and cervical cancer risk: a meta-analysis. Biosci Rep.

[94] Baharvand M, Manifar S, Akkafan R, Mortazavi H, Sabour S (2014). Serum levels of ferritin, copper, and zinc in patients with oral cancer. Biomed J.

[95] Mazdak H, Yazdekhasti F, Movahedian A, Mirkheshti N, Shafieian M (2010). The comparative study of serum iron, copper, and zinc levels between bladder cancer patients and a control group. Int Urol Nephrol.

[96] Lener MR, Scott RJ, Wiechowska-Kozłowska A, Serrano-Fernández P, Baszuk P, Jaworska-Bieniek K (2016). Serum concentrations of selenium and copper in patients diagnosed with pancreatic cancer. Cancer Res Treat.

[97] Ishida S, Andreux P, Poitry-Yamate C, Auwerx J, Hanahan D (2013). Bioavailable copper modulates oxidative phosphorylation and growth of tumors. Proc Natl Acad Sci USA.

[98] Brady DC, Crowe MS, Turski ML, Hobbs GA, Yao X, Chaikuad A (2014). Copper is required for oncogenic BRAF signalling and tumorigenesis. Nature.

[99] Brady DC, Crowe MS, Greenberg DN, Counter CM (2017). Copper chelation inhibits BRAF^V600E^-driven melanomagenesis and counters resistance to BRAF^V600E^ and MEK1/2 inhibitors. Cancer Res.

[100] Grasso M, Bond GJ, Kim YJ, Boyd S, Matson Dzebo M, Valenzuela S (2021). The copper chaperone CCS facilitates copper binding to MEK1/2 to promote kinase activation. J Biol Chem.

[101] Chojnowski JE, Li R, Tsang T, Alfaran FH, Dick A, Cocklin S (2022). Copper modulates the catalytic activity of protein kinase CK2. Front Mol Biosci.

[102] Kimmelman AC, White E (2017). Autophagy and tumor metabolism. Cell Metab.

[103] Tsang T, Posimo JM, Gudiel AA, Cicchini M, Feldser DM, Brady DC (2020). Copper is an essential regulator of the autophagic kinases ULK1/2 to drive lung adenocarcinoma. Nat Cell Biol.

[104] Tsang T, Gu X, Davis CI, Posimo JM, Miller ZA, Brady DC (2022). BRAF^V600E^-driven lung adenocarcinoma requires copper to sustain autophagic signaling and processing. Mol Cancer Res.

[105] Li Y, Chen Y (2019). AMPK and autophagy. Adv Exp Med Biol.

[106] Liao J, Yang F, Yu W, Qiao N, Zhang H, Han Q (2020). Copper induces energy metabolic dysfunction and AMPK-mTOR pathway-mediated autophagy in kidney of broiler chickens. Ecotoxicol Environ Saf.

[107] Xia F, Fu Y, Xie H, Chen Y, Fang D, Zhang W (2022). Suppression of ATG4B by copper inhibits autophagy and involves in Mallory body formation. Redox Biol.

[108] Yu Z, Zhou R, Zhao Y, Pan Y, Liang H, Zhang JS (2019). Blockage of SLC31A1-dependent copper absorption increases pancreatic cancer cell autophagy to resist cell death. Cell Prolif.

[109] Lugano R, Ramachandran M, Dimberg A (2020). Tumor angiogenesis: causes, consequences, challenges and opportunities. Cell Mol Life Sci.

[110] McAuslan BR, Reilly W (1980). Endothelial cell phagokinesis in response to specific metal ions. Exp Cell Res.

[111] Narayanan G, Bharathidevi SR, Vuyyuru H, Muthuvel B, Konerirajapuram NS (2013). CTR1 silencing inhibits angiogenesis by limiting copper entry into endothelial cells. PLoS ONE.

[112] Rigiracciolo DC, Scarpelli A, Lappano R, Pisano A, Santolla MF, De Marco P (2015). Copper activates HIF-1α/GPER/VEGF signalling in cancer cells. Oncotarget.

[113] Sivaraja V, Kumar TK, Rajalingam D, Graziani I, Prudovsky I, Yu C (2006). Copper binding affinity of S100A13, a key component of the FGF-1 nonclassical copper-dependent release complex. Biophys J.

[114] Mandinov L, Mandinova A, Kyurkchiev S, Kyurkchiev D, Kehayov I, Kolev V (2003). Copper chelation represses the vascular response to injury. Proc Natl Acad Sci USA.

[115] Pan Q, Kleer CG, Golen KL, Irani J, Bottema KM, Bias C (2002). Copper deficiency induced by tetrathiomolybdate suppresses tumor growth and angiogenesis. Cancer Res.

[116] Kohno T, Urao N, Ashino T, Sudhahar V, McKinney RD, Hamakubo T (2013). Novel role of copper transport protein antioxidant-1 in neointimal formation after vascular injury. Arterioscler Thromb Vasc Biol.

[117] Chen GF, Sudhahar V, Youn SW, Das A, Cho J, Kamiya T (2015). Copper transport protein antioxidant-1 promotes inflammatory neovascularization via chaperone and transcription factor function. Sci Rep.

[118] Nagaraju GP, Dontula R, El-Rayes BF, Lakka SS (2014). Molecular mechanisms underlying the divergent roles of SPARC in human carcinogenesis. Carcinogenesis.

[119] Blockhuys S, Wittung-Stafshede P (2017). Copper chaperone Atox1 plays role in breast cancer cell migration. Biochem Biophys Res Commun.

[120] Blockhuys S, Zhang X, Wittung-Stafshede P (2020). Single-cell tracking demonstrates copper chaperone Atox1 to be required for breast cancer cell migration. Proc Natl Acad Sci USA.

[121] Cheng F, Peng G, Lu Y, Wang K, Ju Q, Ju Y (2022). Relationship between copper and immunity: the potential role of copper in tumor immunity. Front Oncol.

[122] Liao Y, Zhao J, Bulek K, Tang F, Chen X, Cai G (2020). Inflammation mobilizes copper metabolism to promote colon tumorigenesis via an IL-17-STEAP4-XIAP axis. Nat Commun.

[123] Voli F, Valli E, Lerra L, Kimpton K, Saletta F, Giorgi FM (2020). Intratumoral copper modulates PD-L1 expression and influences tumor immune evasion. Cancer Res.

[124] Du C, Guan X, Liu Y, Xu Z, Du X, Li B (2022). Disulfiram/copper induces antitumor activity against gastric cancer cells in vitro and in vivo by inhibiting S6K1 and c-Myc. Cancer Chemother Pharmacol.

[125] Huang X, Hou Y, Weng X, Pang W, Hou L, Liang Y (2021). Diethyldithiocarbamate-copper complex (CuET) inhibits colorectal cancer progression via miR-16-5p and 15b–5p/ALDH1A3/PKM2 axis-mediated aerobic glycolysis pathway. Oncogenesis.

[126] Krishnamoorthy L, Cotruvo JA, Chan J, Kaluarachchi H, Muchenditsi A, Pendyala VS (2016). Copper regulates cyclic-AMP-dependent lipolysis. Nat Chem Biol.

[127] Wang Y, Zhang X, Chen G, Xing Q, Zhu B, Wang X (2023). Integrated analyses reveal the prognostic, immunological features and mechanisms of cuproptosis critical mediator gene FDX1 in KIRC. Genes Immun.

[128] Zhang Z, Zeng X, Wu Y, Liu Y, Zhang X, Song Z (2022). Cuproptosis-related risk score predicts prognosis and characterizes the tumor microenvironment in hepatocellular carcinoma. Front Immunol.

[129] Quan Y, Li W, Yan R, Cheng J, Xu H, Chen L (2023). Tumor cuproptosis and immune infiltration improve survival of patients with hepatocellular carcinoma with a high expression of ferredoxin 1. Front Oncol.

[130] Zhang Y, Dai X, Li Z (2022). Molecular subtypes of cuproptosis regulators and their correlation with clinical prognosis and immune response in glioma. Am J Transl Res.

[131] Yu W, Liu H, Zhang Y, Liu M, Li W, Wang L (2024). Identification of 10 differentially expressed and cuproptosis-related genes in immune infiltration and prognosis of thyroid carcinoma. Cell Mol Biol.

[132] Gao W, He X, Huangfu Q, Xie Y, Chen K, Sun C (2023). A novel cuproptosis-related prognostic gene signature in adrenocortical carcinoma. J Clin Lab Anal.

[133] Wang C, Guo J, Zhang Y, Zhou S, Jiang B (2024). Cuproptosis-related gene FDX1 suppresses the growth and progression of colorectal cancer by retarding EMT progress. Biochem Genet.

[134] Zhang M, Liu X, Wang D, Ruan X, Wang P, Liu L (2023). A novel cuproptosis-related gene signature to predict prognosis in Glioma. BMC Cancer.

[135] Cai Y, He Q, Liu W, Liang Q, Peng B, Li J (2022). Comprehensive analysis of the potential cuproptosis-related biomarker LIAS that regulates prognosis and immunotherapy of pan-cancers. Front Oncol.

[136] Yan C, Niu Y, Ma L, Tian L, Ma J (2022). System analysis based on the cuproptosis-related genes identifies LIPT1 as a novel therapy target for liver hepatocellular carcinoma. J Transl Med.

[137] Li J, Tuo D, Guo G, Gan J (2023). Aberrant expression of cuproptosis related gene LIPT1 is associated with metabolic dysregulation of fatty acid and prognosis in hepatocellular carcinoma. J Cancer Res Clin Oncol.

[138] Deng R, Zhu L, Jiang J, Chen J, Li H (2024). Cuproptosis-related gene LIPT1 as a prognostic indicator in non-small cell lung cancer: Functional involvement and regulation of ATOX1 expression. Biomol Biomed.

[139] Yan X, Zheng W, Xu FS (2024). Identification and validation of a novel cuproptosis signature for stratifying different prognostic, immune, metabolic, and therapeutic landscapes in pancreatic adenocarcinoma. Eur Rev Med Pharmacol Sci.

[140] Xu L, Wu P, Rong A, Li K, Xiao X, Zhang Y (2023). Systematic pan-cancer analysis identifies cuproptosis-related gene DLAT as an immunological and prognostic biomarker. Aging.

[141] Bai WD, Liu JY, Li M, Yang X, Wang YL, Wang GJ (2022). A Novel cuproptosis-related signature identified DLAT as a prognostic biomarker for hepatocellular carcinoma patients. World J Oncol.

[142] Yang Q, Zeng S, Liu W (2023). Roles of cuproptosis-related gene DLAT in various cancers: a bioinformatic analysis and preliminary verification on pro-survival autophagy. PeerJ.

[143] Fang Z, Wang W, Liu Y, Hua J, Liang C, Liu J (2023). Cuproptosis-related gene DLAT as a novel biomarker correlated with prognosis, chemoresistance, and immune infiltration in pancreatic adenocarcinoma: a preliminary study based on bioinformatics analysis. Curr Oncol.

[144] Zhang P, Qiu J, Wang Q, Xu Y, Wang Z, Peng F (2023). DLAT, as a cuproptosis-related gene, regulates kidney renal clear cell carcinoma progression. J Biol Regul Homeost Agents.

[145] Li R, Tong R, Zhang JL, Zhang Z, Deng M, Hou G (2024). Comprehensive molecular analyses of cuproptosis-related genes with regard to prognosis, immune landscape, and response to immune checkpoint blockers in lung adenocarcinoma. J Cancer Res Clin Oncol.

[146] Zhang J, Mao S, Guo Y, Wu Y, Yao X, Huang Y (2019). Inhibition of GLS suppresses proliferation and promotes apoptosis in prostate cancer. Biosci Rep.

[147] Zhao R, Choi BY, Lee MH, Bode AM, Dong Z (2016). Implications of genetic and epigenetic alterations of CDKN2A (p16(INK4a)) in cancer. EBioMedicine.

[148] He J, Jiang X, Yu M, Wang P, Fu L, Zhang G (2023). MTF1 has the potential as a diagnostic and prognostic marker for gastric cancer and is associated with good prognosis. Clin Transl Oncol.

[149] Song L, Zeng R, Yang K, Liu W, Xu Z, Kang F (2023). The biological significance of cuproptosis-key gene MTF1 in pan-cancer and its inhibitory effects on ROS-mediated cell death of liver hepatocellular carcinoma. Discov Oncol.

[150] Yang Y, Qian Cai Q, Sheng FuL, Wei Dong Y, Fan F, Zhong WuX (2022). Reduced N6-methyladenosine mediated by METTL3 acetylation promotes mtf1 expression and hepatocellular carcinoma cell growth. Chem Biodivers.

[151] Li X, Ma Z, Mei L (2022). Cuproptosis-related gene SLC31A1 is a potential predictor for diagnosis, prognosis and therapeutic response of breast cancer. Am J Cancer Res.

[152] Li L, Li L, Sun Q (2022). High expression of cuproptosis-related SLC31A1 gene in relation to unfavorable outcome and deregulated immune cell infiltration in breast cancer: an analysis based on public databases. BMC Bioinf.

[153] Lian W, Yang P, Li L, Chen D, Wang C (2023). A ceRNA network-mediated over-expression of cuproptosis-related gene SLC31A1 correlates with poor prognosis and positive immune infiltration in breast cancer. Front Med (Lausanne).

[154] Kong FS, Ren CY, Jia R, Zhou Y, Chen JH, Ma Y (2023). Systematic pan-cancer analysis identifies SLC31A1 as a biomarker in multiple tumor types. BMC Med Genomics.

[155] Zhang G, Wang N, Ma S, Tao P, Cai H (2024). Comprehensive analysis of the effects of the cuprotosis-associated gene SLC31A1 on patient prognosis and tumor microenvironment in human cancer. Transl Cancer Res.

[156] Lin YZ, Liu WH, Wu YP, Cai H, Zheng QS, Wei Y (2024). Revealing the potential of solute carrier family 31 (copper transporters), member 1: Insights into its role in bladder cancer progression and therapeutic implications. Int J Immunopathol Pharmacol.

[157] Zhu J, Wang J, Liu H, Lei T, Yang J, Lan S (2023). Crosstalk of cuproptosis-related prognostic signature and competing endogenous RNAs regulation in hepatocellular carcinoma. Aging.

[158] Yuan D, Li XQ, Qu FW, Wang Y (2023). Landscape and the immune patterns of cuproptosis in oral squamous cell carcinoma. J Oral Pathol Med.

[159] Li S, Weng J, Xiao C, Lu J, Cao W, Song F (2023). Cuproptosis-related molecular patterns and gene (ATP7A) in hepatocellular carcinoma and their relationships with tumor immune microenvironment and clinical features. Cancer Rep.

[160] Zhang Z, Ma Y, Guo X, Du Y, Zhu Q, Wang X (2021). FDX1 can impact the prognosis and mediate the metabolism of lung adenocarcinoma. Front Pharmacol.

[161] Guowei L, Xiufang L, Qianqian X, Yanping J (2023). The FDX1 methylation regulatory mechanism in the malignant phenotype of glioma. Genomics.

[162] Solmonson A, DeBerardinis RJ (2018). Lipoic acid metabolism and mitochondrial redox regulation. J Biol Chem.

[163] Chen Q, Wang Y, Yang L, Sun L, Wen Y, Huang Y (2022). PM2.5 promotes NSCLC carcinogenesis through translationally and transcriptionally activating DLAT-mediated glycolysis reprograming. J Exp Clin Cancer Res.

[164] Bian Z, Fan R, Xie L (2022). A novel cuproptosis-related prognostic gene signature and validation of differential expression in clear cell renal cell carcinoma. Genes.

[165] Martínez-Reyes I, Chandel NS (2020). Mitochondrial TCA cycle metabolites control physiology and disease. Nat Commun.

[166] Szeliga M, Bogacińska-Karaś M, Różycka A, Hilgier W, Marquez J, Albrecht J (2014). Silencing of GLS and overexpression of GLS2 genes cooperate in decreasing the proliferation and viability of glioblastoma cells. Tumour Biol.

[167] Lu R, Zhang X, Li X, Wan X (2020). Circ_0016418 promotes melanoma development and glutamine catabolism by regulating the miR-605-5p/GLS axis. Int J Clin Exp Pathol.

[168] Huang Q, Lian C, Dong Y, Zeng H, Liu B, Xu N (2021). SNAP25 inhibits glioma progression by regulating synapse plasticity via GLS-mediated glutaminolysis. Front Oncol.

[169] Mukha A, Kahya U, Linge A, Chen O, Löck S, Lukiyanchuk V (2021). GLS-driven glutamine catabolism contributes to prostate cancer radiosensitivity by regulating the redox state, stemness and ATG5-mediated autophagy. Theranostics.

[170] Tam KW, Zhang W, Soh J, Stastny V, Chen M, Sun H (2013). CDKN2A/p16 inactivation mechanisms and their relationship to smoke exposure and molecular features in non-small-cell lung cancer. J Thorac Oncol.

[171] Cousins RJ (1985). Absorption, transport, and hepatic metabolism of copper and zinc: special reference to metallothionein and ceruloplasmin. Physiol Rev.

[172] Zhang R, Zhao G, Shi H, Zhao X, Wang B, Dong P (2020). Zinc regulates primary ovarian tumor growth and metastasis through the epithelial to mesenchymal transition. Free Radic Biol Med.

[173] Franklin RB, Costello LC (2007). Zinc as an anti-tumor agent in prostate cancer and in other cancers. Arch Biochem Biophys.

[174] Costello LC, Franklin RB (2011). Zinc is decreased in prostate cancer: an established relationship of prostate cancer!. J Biol Inorg Chem.

[175] Han H, Nakaoka HJ, Hofmann L, Zhou JJ, Yu C, Zeng L (2022). The Hippo pathway kinases LATS1 and LATS2 attenuate cellular responses to heavy metals through phosphorylating MTF1. Nat Cell Biol.

[176] Jing J, Ma M, Yan B, Qiu B, Lu S, Yang L (2022). A SLC31A1-MEK-DNMT1-miR-124 feedback loop contributes to pancreatic cancer progression. Genes Dis.

[177] Inoue Y, Matsumoto H, Yamada S, Kawai K, Suemizu H, Gika M (2010). Association of ATP7A expression and in vitro sensitivity to cisplatin in non-small cell lung cancer. Oncol Lett.

[178] Higashimoto M, Kanzaki A, Shimakawa T, Konno S, Naritaka Y, Nitta Y (2003). Expression of copper-transporting P-type adenosine triphosphatase in human esophageal carcinoma. Int J Mol Med.

[179] Li ZH, Qiu MZ, Zeng ZL, Luo HY, Wu WJ, Wang F (2012). Copper-transporting P-type adenosine triphosphatase (ATP7A) is associated with platinum-resistance in non-small cell lung cancer (NSCLC). J Transl Med.

[180] Huang X, Zhou S, Tóth J, Hajdu A (2022). Cuproptosis-related gene index: a predictor for pancreatic cancer prognosis, immunotherapy efficacy, and chemosensitivity. Front Immunol.

[181] Zhang B, Wang Q, Zhang T, Zheng Z, Lin Z, Zhou S (2023). Identification and validation of a novel cuproptosis-related gene signature in multiple myeloma. Front Cell Dev Biol.

[182] Yao K, Zhang R, Li L, Liu M, Feng S, Yan H (2023). The signature of cuproptosis-related immune genes predicts the tumor microenvironment and prognosis of prostate adenocarcinoma. Front Immunol.

[183] Du Y, Lin Y, Wang B, Li Y, Xu D, Gan L (2022). Cuproptosis patterns and tumor immune infiltration characterization in colorectal cancer. Front Genet.

[184] Huang Y, Yin D, Wu L (2022). Identification of cuproptosis-related subtypes and development of a prognostic signature in colorectal cancer. Sci Rep.

[185] Wu W, Dong J, Lv Y, Chang D (2022). Cuproptosis-Related genes in the prognosis of colorectal cancer and their correlation with the tumor microenvironment. Front Genet.

[186] Huang H, Long Z, Xie Y, Qin P, Kuang L, Li X (2022). Molecular subtypes based on cuproptosis-related genes and tumor microenvironment infiltration characterization in colorectal cancer. J Oncol.

[187] Paterson BM, Donnelly PS (2011). Copper complexes of bis(thiosemicarbazones): from chemotherapeutics to diagnostic and therapeutic radiopharmaceuticals. Chem Soc Rev.

[188] Cater MA, Pearson HB, Wolyniec K, Klaver P, Bilandzic M, Paterson BM (2013). Increasing intracellular bioavailable copper selectively targets prostate cancer cells. ACS Chem Biol.

[189] O'Day SJ, Eggermont AM, Chiarion-Sileni V, Kefford R, Grob JJ, Mortier L (2013). Final results of phase III SYMMETRY study: randomized, double-blind trial of elesclomol plus paclitaxel versus paclitaxel alone as treatment for chemotherapy-naive patients with advanced melanoma. J Clin Oncol.

[190] Dong Z, Cui H (2018). The autophagy-lysosomal pathways and their emerging roles in modulating proteostasis in tumors. Cells.

[191] Orlowski RZ, Kuhn DJ (2008). Proteasome inhibitors in cancer therapy: lessons from the first decade. Clin Cancer Res.

[192] Tsvetkov P, Detappe A, Cai K, Keys HR, Brune Z, Ying W (2019). Mitochondrial metabolism promotes adaptation to proteotoxic stress. Nat Chem Biol.

[193] Corazao-Rozas P, Guerreschi P, Jendoubi M, André F, Jonneaux A, Scalbert C (2013). Mitochondrial oxidative stress is the Achille’s heel of melanoma cells resistant to Braf-mutant inhibitor. Oncotarget.

[194] Wen H, Qu C, Wang Z, Gao H, Liu W, Wang H (2023). Cuproptosis enhances docetaxel chemosensitivity by inhibiting autophagy via the DLAT/mTOR pathway in prostate cancer. FASEB J.

[195] Silva-Rodríguez P, Fernández-Díaz D, Bande M, Pardo M, Loidi L, Blanco-Teijeiro MJ (2022). GNAQ and GNA11 genes: a comprehensive review on oncogenesis, prognosis and therapeutic opportunities in uveal melanoma. Cancers.

[196] Li Y, Yang J, Zhang Q, Xu S, Sun W, Ge S (2022). Copper ionophore elesclomol selectively targets GNAQ/11-mutant uveal melanoma. Oncogene.

[197] Li H, Wang J, Wu C, Wang L, Chen ZS, Cui W (2020). The combination of disulfiram and copper for cancer treatment. Drug Discov Today.

[198] Skrott Z, Mistrik M, Andersen KK, Friis S, Majera D, Gursky J (2017). Alcohol-abuse drug disulfiram targets cancer via p97 segregase adaptor NPL4. Nature.

[199] Chen C, Nie D, Huang Y, Yu X, Chen Z, Zhong M (2022). Anticancer effects of disulfiram in T-cell malignancies through NPL4-mediated ubiquitin-proteasome pathway. J Leukoc Biol.

[200] Skrott Z, Majera D, Gursky J, Buchtova T, Hajduch M, Mistrik M (2019). Disulfiram's anti-cancer activity reflects targeting NPL4, not inhibition of aldehyde dehydrogenase. Oncogene.

[201] Yang X, Deng L, Diao X, Yang S, Zou L, Yang Q (2023). Targeting cuproptosis by zinc pyrithione in triple-negative breast cancer. iScience.

[202] Nie X, Chen H, Xiong Y, Chen J, Liu T (2022). Anisomycin has a potential toxicity of promoting cuproptosis in human ovarian cancer stem cells by attenuating YY1/lipoic acid pathway activation. J Cancer.

[203] Yang W, Wang Y, Huang Y, Yu J, Wang T, Li C (2023). 4-Octyl itaconate inhibits aerobic glycolysis by targeting GAPDH to promote cuproptosis in colorectal cancer. Biomed Pharmacother.

[204] Cheng Z, Li M, Dey R, Chen Y (2021). Nanomaterials for cancer therapy: current progress and perspectives. J Hematol Oncol.

[205] Xu Y, Liu SY, Zeng L, Ma H, Zhang Y, Yang H (2022). An enzyme-engineered nonporous copper(I) coordination polymer nanoplatform for cuproptosis-based synergistic cancer therapy. Adv Mater.

[206] Wu W, Yu L, Jiang Q, Huo M, Lin H, Wang L (2019). Enhanced tumor-specific disulfiram chemotherapy by in situ Cu^2+^ chelation-initiated nontoxicity-to-toxicity transition. J Am Chem Soc.

[207] Lu S, Tian H, Li B, Li L, Jiang H, Gao Y (2024). An ellagic acid coordinated copper-based nanoplatform for efficiently overcoming cancer chemoresistance by cuproptosis and synergistic inhibition of cancer cell stemness. Small.

[208] Xia Y, Gu M, Wang J, Zhang X, Shen T, Shi X (2024). Tumor microenvironment-activated, immunomodulatory nanosheets loaded with copper(II) and 5-FU for synergistic chemodynamic therapy and chemotherapy. J Coll Interface Sci.

[209] Pashootan P, Saadati F, Fahimi H, Rahmati M, Strippoli R, Zarrabi A (2024). Metal-based nanoparticles in cancer therapy: exploring photodynamic therapy and its interplay with regulated cell death pathways. Int J Pharm.

[210] Dong C, Feng W, Xu W, Yu L, Xiang H, Chen Y (2020). The coppery age: copper (cu)-involved nanotheranostics. Adv Sci.

[211] Ning S, Lyu M, Zhu D, Lam JWY, Huang Q, Zhang T (2023). Type-I AIE photosensitizer loaded biomimetic system boosting cuproptosis to inhibit breast cancer metastasis and rechallenge. ACS Nano.

[212] Zhou J, Yu Q, Song J, Li S, Li XL, Kang BK (2023). Photothermally triggered copper payload release for cuproptosis-promoted cancer synergistic therapy. Angew Chem Int Ed Engl.

[213] Zhang X, Zhu J, Wang S, Li S, Jiaoting E, Hu J (2024). A Copper/Ferrous-engineering redox homeostasis disruptor for cuproptosis/ferroptosis co-activated nanocatalytic therapy in liver cancer. Adv Funct Mater.

[214] Guo B, Yang F, Zhang L, Zhao Q, Wang W, Yin L (2023). Cuproptosis induced by ROS responsive nanoparticles with elesclomol and copper combined with αPD-L1 for enhanced cancer immunotherapy. Adv Mater.

[215] Tian H, Duan J, Li B, Qin S, Nice EC, Zhang W (2023). Clinical chemotherapeutic agent coordinated copper-based nanoadjuvants for efficiently sensitizing cancer chemo-immunotherapy by cuproptosis-mediated mitochondrial metabolic reprogramming. Adv Funct Mater.

[216] Jin XK, Liang JL, Zhang SM, Huang QX, Zhang SK, Liu CJ (2023). Orchestrated copper-based nanoreactor for remodeling tumor microenvironment to amplify cuproptosis-mediated anti-tumor immunity in colorectal cancer. Mater Today.

[217] Huang QX, Liang JL, Chen QW, Jin XK, Niu MT, Dong CY (2023). Metal-organic framework nanoagent induces cuproptosis for effective immunotherapy of malignant glioblastoma. Nano Today.

[218] Hu P, Li Y, Zhang L, Lan X, Ren X, Liang W (2024). Defect-engineered photothermal nanozyme with NIR-II absorption induces cuproptosis-apoptosis for synergized cancer immunotherapy and fast wound healing. Mater Des.

[219] Yan C, Lv H, Feng Y, Li Y, Zhao Z (2024). Inhalable nanoparticles with enhanced cuproptosis and cGAS–STING activation for synergistic lung metastasis immunotherapy. Acta Pharm Sin B.

[220] Li W, Xiao Y, Guo G, Peng J, Zhu N, Chen Z (2024). Cuprous oxide nanocomposites with photothermal (PTT) and chemical dynamics (CDT) effects induce cuproptosis in breast cancer using the strategy of increasing inflow and reducing outflow. Nano Today.

[221] Zhang J, Peng L, Hao Y, Yang H, Zhao W, Mao C (2023). Biodegradable CuMoO_4_ nanodots with multienzyme activities for multimodal treatment of tumor. Adv Healthc Mater.

[222] Ni C, Ouyang Z, Li G, Liu J, Cao X, Zheng L (2023). A tumor microenvironment-responsive core-shell tecto dendrimer nanoplatform for magnetic resonance imaging-guided and cuproptosis-promoted chemo-chemodynamic therapy. Acta Biomater.

[223] Yang Z, Zhao Z, Cheng H, Shen Y, Xie A, Zhu M (2023). In-situ fabrication of novel Au nanoclusters-Cu^2+^@sodium alginate/hyaluronic acid nanohybrid gels for cuproptosis enhanced photothermal/photodynamic/chemodynamic therapy via tumor microenvironment regulation. J Coll Interface Sci.

[224] Zhong J, Zheng X, Wen Y, Wang SB, Zhan G, Chen AZ (2023). In situ sacrificial growth of metastable copper-enriched nanomedicine for cuproptosis-based synergistic cancer therapy. Chem Eng J.

[225] Chen K, Zhou A, Zhou X, Liu Y, Xu Y, Ning X (2023). An intelligent cell-derived nanorobot bridges synergistic crosstalk between sonodynamic therapy and cuproptosis to promote cancer treatment. Nano Lett.

[226] Liu T, Zhou Z, Zhang M, Lang P, Li J, Liu Z (2023). Cuproptosis-immunotherapy using PD-1 overexpressing T cell membrane-coated nanosheets efficiently treats tumor. J Control Release.

[227] Chan L, Liu Y, Chen M, Su Y, Guo J, Zhu L (2023). Cuproptosis-driven enhancement of thermotherapy by sequentially response cu_2-x_se via copper chemical transition. Adv Funct Mater.

[228] Xu W, Wang Y, Hou G, Wang J, Wang T, Qian J (2023). Tumor microenvironment responsive hollow nanoplatform for triple amplification of oxidative stress to enhance cuproptosis-based synergistic cancer therapy. Adv Healthc Mater.

[229] Yu Q, Zhou J, Liu Y, Li XQ, Li S, Zhou H (2023). DNAzyme-mediated cascade nanoreactor for cuproptosis-promoted pancreatic cancer synergistic therapy. Adv Healthc Mater.

[230] Lu Y, Pan Q, Gao W, Pu Y, He B (2022). Reversal of cisplatin chemotherapy resistance by glutathione-resistant copper-based nanomedicine via cuproptosis. J Mater Chem B.

[231] Zhang J, Han M, Zhang J, Abdalla M, Sun P, Yang Z (2023). Syphilis mimetic nanoparticles for cuproptosis-based synergistic cancer therapy via reprogramming copper metabolism. Int J Pharm.

[232] Zhao F, Yu H, Liang L, Wang C, Shi D, Zhang X (2023). Redox homeostasis disruptors based on metal-phenolic network nanoparticles for chemo/chemodynamic synergistic tumor therapy through activating apoptosis and cuproptosis. Adv Healthc Mater.

[233] Jia W, Tian H, Jiang J, Zhou L, Li L, Luo M (2023). Brain-targeted HFn-Cu-REGO nanoplatform for site-specific delivery and manipulation of autophagy and cuproptosis in glioblastoma. Small.

[234] Zhao F, Liang L, Wang H, Wang C, Su D, Ying Y (2023). H_2_S-activated ion-interference therapy: a novel tumor targeted therapy based on copper-overload-mediated cuproptosis and pyroptosis. Adv Funct Mater.

[235] Zhu G, Wang M, Qiao L, Xie Y, Wang J, Li L (2024). Lysosomal rupture-mediated “broken window effect” to amplify cuproptosis and pyroptosis for high-efficiency cancer immunotherapy. Adv Funct Mater.

[236] Liu Y, Niu R, Zhao H, Wang Y, Song S, Zhang H (2024). Single-site nanozymes with a highly conjugated coordination structure for antitumor immunotherapy via cuproptosis and cascade-enhanced T lymphocyte activity. J Am Chem Soc.

[237] Yan C, Liu Y, Zhao G, Yang H, Lv H, Li G (2024). Inhalable metal-organic framework-mediated cuproptosis combined with PD-L1 checkpoint blockade for lung metastasis synergistic immunotherapy. Acta Pharm Sin B.

[238] Ye L, Yu C, Xia J, Ni K, Zhang Y, Ying X (2024). Multifunctional nanomaterials via cell cuproptosis and oxidative stress for treating osteosarcoma and OS-induced bone destruction. Mater Today Bio.

[239] Xiao C, Li J, Hua A, Wang X, Li S (2024). Hyperbaric oxygen boosts antitumor efficacy of copper-diethyldithiocarbamate nanoparticles against pancreatic ductal adenocarcinoma by regulating cancer stem cell metabolism. Research.

[240] Dai Y, Zhu L, Li X, Zhang F, Chen K, Jiao G (2024). A biomimetic cuproptosis amplifier for targeted NIR-II fluorescence/photoacoustic imaging-guided synergistic NIR-II photothermal immunotherapy. Biomaterials.

[241] Hao C, Huang L, Zhang H, Xu L, Sun M, Kuang H (2023). Chiral CuxOS@ Fe-MOFs for enhanced cancer therapy. Adv Funct Mater.

[242] Xia J, Hu C, Ji Y, Wang M, Jin Y, Ye L (2023). Copper-loaded nanoheterojunction enables superb orthotopic osteosarcoma therapy via oxidative stress and cell cuproptosis. ACS Nano.

[243] Qiao L, Zhu G, Jiang T, Qian Y, Sun Q, Zhao G (2024). Self-destructive copper carriers induce pyroptosis and cuproptosis for efficient tumor immunotherapy against dormant and recurrent tumors. Adv Mater.

[244] Chen P, Liu XQ, Lin X, Gao LY, Zhang S, Huang X (2021). Targeting YTHDF1 effectively re-sensitizes cisplatin-resistant colon cancer cells by modulating GLS-mediated glutamine metabolism. Mol Ther Oncol.

[245] Harrach S, Ciarimboli G (2015). Role of transporters in the distribution of platinum-based drugs. Front Pharmacol.

[246] Wu G, Peng H, Tang M, Yang M, Wang J, Hu Y (2021). ZNF711 down-regulation promotes CISPLATIN resistance in epithelial ovarian cancer via interacting with JHDM2A and suppressing SLC31A1 expression. EBioMedicine.

[247] Cheng C, Ding Q, Zhang Z, Wang S, Zhong B, Huang X (2020). PTBP1 modulates osteosarcoma chemoresistance to cisplatin by regulating the expression of the copper transporter SLC31A1. J Cell Mol Med.

[248] Chisholm CL, Wang H, Wong AH, Vazquez-Ortiz G, Chen W, Xu X (2016). Ammonium tetrathiomolybdate treatment targets the copper transporter ATP7A and enhances sensitivity of breast cancer to cisplatin. Oncotarget.

[249] Yu Z, Cao W, Ren Y, Zhang Q, Liu J (2020). ATPase copper transporter A, negatively regulated by miR-148a-3p, contributes to cisplatin resistance in breast cancer cells. Clin Transl Med.

[250] Petruzzelli R, Mariniello M, De Cegli R, Catalano F, Guida F, Di Schiavi E (2022). TFEB Regulates ATP7B expression to promote platinum chemoresistance in human ovarian cancer cells. Cells.

[251] Katano K, Safaei R, Samimi G, Holzer A, Rochdi M, Howell SB (2003). The copper export pump ATP7B modulates the cellular pharmacology of carboplatin in ovarian carcinoma cells. Mol Pharmacol.

[252] Kalayda GV, Wagner CH, Buss I, Reedijk J, Jaehde U (2008). Altered localisation of the copper efflux transporters ATP7A and ATP7B associated with cisplatin resistance in human ovarian carcinoma cells. BMC Cancer.

[253] Janardhanan P, Somasundaran AK, Balakrishnan AJ, Pilankatta R (2022). Sensitization of cancer cells towards Cisplatin and Carboplatin by protein kinase D inhibitors through modulation of ATP7A/B (copper transport ATPases). Cancer Treat Res Commun.

[254] Ryumon S, Okui T, Kunisada Y, Kishimoto K, Shimo T, Hasegawa K (2019). Ammonium tetrathiomolybdate enhances the antitumor effect of cisplatin via the suppression of ATPase copper transporting beta in head and neck squamous cell carcinoma. Oncol Rep.

[255] Cao HZ, Yang WT, Zheng PS (2022). Cytotoxic effect of disulfiram/copper on human cervical cancer cell lines and LGR5-positive cancer stem-like cells. BMC Cancer.

[256] Kita Y, Hamada A, Saito R, Teramoto Y, Tanaka R, Takano K (2019). Systematic chemical screening identifies disulfiram as a repurposed drug that enhances sensitivity to cisplatin in bladder cancer: a summary of preclinical studies. Br J Cancer.

